# Impact of *CHEK2* germline variants on haematological malignancy risk and outcomes of allogeneic HSCT

**DOI:** 10.1111/bjh.20196

**Published:** 2025-06-12

**Authors:** Atte K. Lahtinen, Maarja Karu, Jessica R. Koski, Jarmo Ritari, Kati Hyvärinen, Satu Koskela, Julia Nihtilä, Jukka Partanen, Kim Vettenranta, Minna Koskenvuo, Riitta Niittyvuopio, Urpu Salmenniemi, Maija Itälä‐Remes, Kirsi Jahnukainen, Outi Kilpivaara, Ulla Wartiovaara‐Kautto

**Affiliations:** ^1^ Applied Tumor Genomics Research Program, Faculty of Medicine University of Helsinki Helsinki Finland; ^2^ Department of Medical and Clinical Genetics, Medicum, Faculty of Medicine University of Helsinki Helsinki Finland; ^3^ Department of Hematology and Oncology Tallinn Children's Hospital Tallinn Estonia; ^4^ New Children's Hospital, Pediatric Research Center University of Helsinki and Helsinki University Hospital Helsinki Finland; ^5^ Research and Development Finnish Red Cross Blood Service Helsinki Finland; ^6^ Department of Hematology, Comprehensive Cancer Center Helsinki University Hospital, and University of Helsinki Helsinki Finland; ^7^ Finnish Medicines Agency Helsinki Finland; ^8^ Department of Clinical Hematology and Stem Cell Transplant Unit Turku University Hospital, University of Turku Turku Finland; ^9^ Department of Women's and Children's Health, NORDFERTIL Research Lab Stockholm Karolinska Institutet and University Hospital Stockholm Sweden; ^10^ HUSLAB Laboratory of Genetics, HUS Diagnostic Center Helsinki University Hospital Helsinki Finland; ^11^ K. Albin Johansson Cancer Research Fellow Foundation for the Finnish Cancer Institute Helsinki Finland

**Keywords:** acute leukaemia, allogeneic haematopoietic stem cell transplantation, *CHEK2*, germline, haematological malignancy

To the Editor,

The significance of germline genetics in haematological malignancies (HM) is increasingly acknowledged. This has led to the development of clinical guidelines for recognizing the risk of hereditary HMs and in selected patients tailoring the conditioning and graft‐versus‐host disease (GVHD) prophylaxis undergoing haematopoietic stem cell transplantation (HSCT).^1^, ^2^, ^3^, ^4^, ^5^, ^6^, ^7^

Today, more than 100 genes associated with increased risk for HM are acknowledged and included in the germline screening panels. Predisposition to cancer is most often caused by aberrations in genes involved in key cellular functions, such as DNA damage repair (DDR). Variants in *CHEK2*, a DDR gene, predispose to solid cancer, particularly breast cancer.^8^, ^9^, ^10^ Recent data suggest that rare germline *CHEK2* variants may also affect the risk of myeloid HMs.^11^, ^12^ Furthermore, we have observed an increased risk for acute lymphoblastic leukaemia.^13^

The most studied *CHEK2* variants globally, (NM_007194.4) c.1229del, p. Cys410SerfsTer4 and c.470T>C, p. Ile157Thr, show a markedly higher prevalence in the Finnish population (0.86% and 2.58%, respectively) compared to non‐Finnish populations (0.19% and 0.18%, respectively).^14^ The ascending role of *CHEK2* variants in HM predisposition and the lack of reports on their impact on the outcomes of HSCTs prompted us to perform this study.

The patients and analysis methods are described in Figure 1A; Supporting Information and Table S1. We analysed data from adult and paediatric patients who underwent HSCT (*n* = 877), along with a subset of paired HSCT donors (*n* = 669). The study cohort comprised three adult and one paediatric cohort (Figure 1A). We used whole exome sequencing (WES) data of patient samples and analysed germline variants with a panel of genes predisposing to haematological malignancies (haematology panel) and solid cancers (oncology panel) as previously described in Lahtinen et al^4^ (Table S2). To validate our findings, we used an independent HSCT cohort of patients with single nucleotide polymorphism (SNP) array data covering the *CHEK2* c.1229del and c.470T>C (Figure 1A: Adult cohort 3). Furthermore, we used WES and SNP array data available for the respective HSCT donors (Figure 1A; Table S3). During data analysis, we focused on variants that can be reliably analysed using WES and SNP methods. However, we acknowledge that the detection of intronic variants is a limitation of our study design.

**FIGURE 1:**
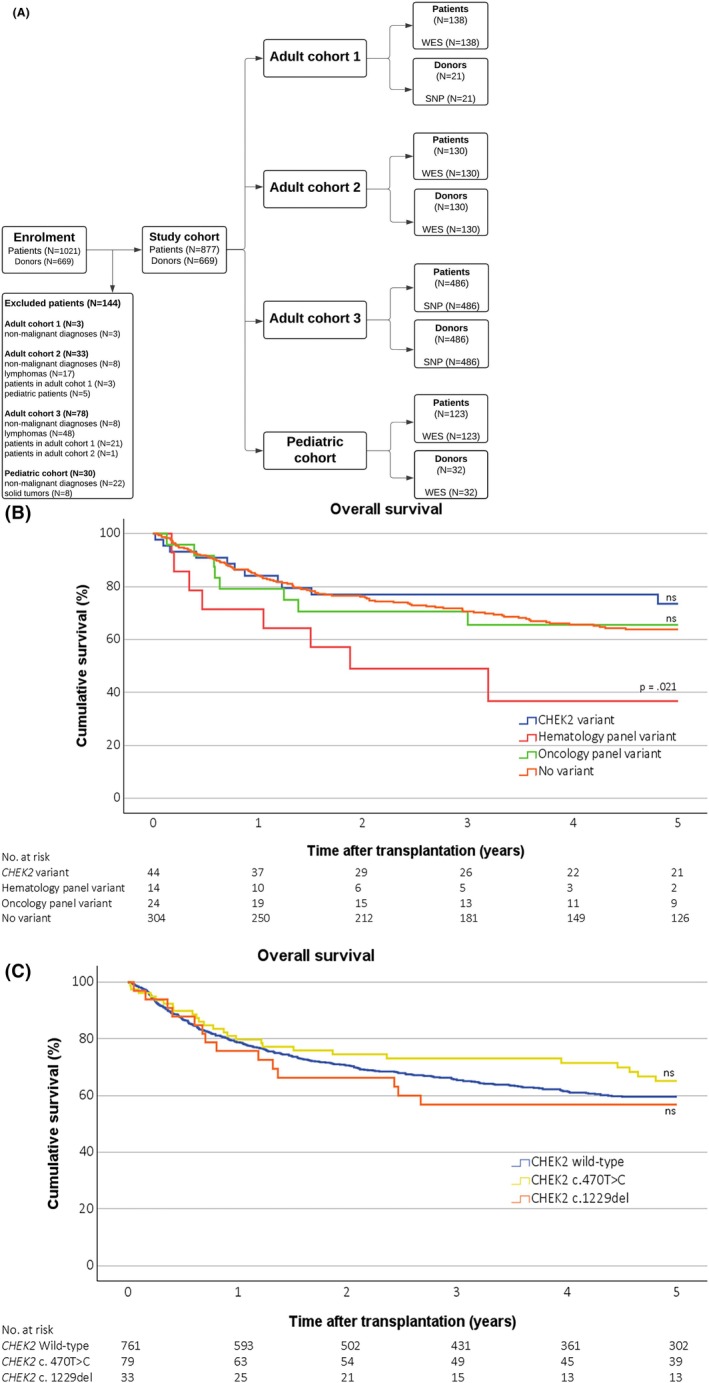
Flowchart of the study cohort and overall survival by variant status. (A) The flowchart depicts the formation of the study cohort with respective genetic data included. (B) Overall survival is calculated for recipients in Adult cohort 1, Adult cohort 2 and Pediatric cohort by WES data and (C) in all four study cohorts by WES and SNP data. The Kaplan–Meier curve comparing (B) recipients with and without *CHEK2*, haematology panel, oncology panel or (C) *CHEK2* c.1229del, p.Cys410SerfsTer4 and c.470T>C, p.Ile157Thr variants. Recipients with a haematology gene variant showed significantly reduced overall survival compared to those without any variant, as indicated by the log‐rank test *p*‐value. There were no significant differences in overall survival in patients with (B) the oncology panel or *CHEK2* variant or (C) the *CHEK2* c.1229del or c.470T>C variant compared to those without these variants. Five patients carrying multiple gene variants were excluded from the analysis in the WES dataset and three patients carrying both *CHEK2* variants were excluded from the SNP dataset. Tick marks indicate censored data. Ns: Not significant; SNP: Single nucleotide polymorphism; WES: Whole exome sequencing.

We analysed overall survival (OS), relapse rates and non‐relapse mortality (NRM) using Kaplan–Meier estimations and log‐rank tests. Five patients of 391 with multiple variants in the WES data and three patients carrying two *CHEK2* variants identified in the SNP cohort were excluded from the analyses. Differences in minor allele frequencies (MAF) between patients, donors and the gnomAD Finnish non‐cancer controls (gnomAD v4.1.0 Finns, *n* = 32 026)^14^ were assessed using two‐sided Fisher's exact tests. Given the genetic isolation of the Finnish population, gnomAD provides an adequately powered reference cohort for comparison. The 877 patient samples provided at least 80% power to detect a twofold increased risk of HMs associated with *CHEK2* variants.

Germline pathogenic (P) or likely pathogenic (LP) variants were identified in 3.6% (haematology panel, *n* = 14/386) and 6.2% (oncology panel, *n* = 24/386) of the patients in the WES dataset (Table S4). The most prevalent P/LP variants found in the genes included in the panels were in *CHEK2*. Thus, we also included *CHEK2* c.470C>T in further analyses considering its association with cancer risk, despite having conflicting classifications of pathogenicity.^13^ Clinical parameters did not significantly differ between the *CHEK2* variant, haematology panel and oncology panel variant carriers (Table S5). The proportion of family donors was slightly lower in patients with a gene variant included in the haematology panel compared to the *CHEK2* variant, oncology panel variant and no variant groups (35.7% vs. 56.8%, 58.3% and 52.3%, respectively) likely reflecting the inherited nature of the disease (Table S5).

The median follow‐up time after HSCT was 46 months (range: 0–258 months). The OS, relapse rate and NRM among patients with the *CHEK2* or oncology panel variants did not differ from those without an identified variant. In contrast, patients carrying P/LP variants included in the haematology panel had a significantly lower OS (*p* = 0.021) compared to patients without identified germline variants (Figure 1B). The difference in the OS resulted from a combination of excess NRM and increased relapse rate, but these findings were not significant (Figure S1). Table S6 presents demographic data, transplant‐related characteristics and the outcome of patients carrying a P/LP variant in the haematology panel. We did not detect differences in rates of acute (severe or all stages) or chronic (extensive or all stages) GVHD between any of the groups (Figure S2).

When analysing the SNP cohort, we found that 4.9% (*n* = 24/486) and 9.7% (*n* = 47/486) of patients carried c.1229del and c.470C>T, respectively. We then combined the WES and SNP datasets for further analyses. In total, we identified the *CHEK2* c.1229del variant in 36/877 (4.1%; 2 homozygous and 34 heterozygous) and c.470T>C in 82/877 (9.4%; 5 homozygous and 77 heterozygous) patients (Table 1). The MAFs were significantly higher in patients than in the gnomAD Finnish population control group: 0.022 versus 0.009 (c.1229del, *p* < 0.001) and 0.050 vs. 0.025 (c.470T>C, *p* < 0.001). Remarkably, the odds ratio for our patients with HM was 2.5 (95% CI, 1.7 to 3.5) and 1.9 (95% CI, 1.5 to 2.4) for c.1229del and c.470T>C respectively (Table 1). Among acute leukaemia patients, the prevalence of the *CHEK2* c.1229del variant was similar in paediatric and adult groups (OR 2.73, 95% CI 1.11–6.73; and OR 2.54, 95% CI: 1.57–4.10 respectively). In contrast, the MAF of the c.470C>T variant was lower in paediatric patients (OR 1.27, 95% CI 0.59–2.73) compared to adults (OR 2.15, 95% CI: 1.57–2.95), suggesting a potentially weaker association in children. Due to the limited paediatric sample size, we refrain from making strong conclusions.

**TABLE 1:** Enrichment of *CHEK2* variants c.1229del, p.Cys410SerfsTer4 and c.470T>C, p.Ile157Thr in a cohort of 877 Finnish individuals with haematological malignancies, compared to their prevalence in the general Finnish population according to gnomAD v4.1.0.

*CHEK2* c.1229del, p.Cys410SerfsTer4	Hom	Het	WT	Total	MAF	OR (95% CI)	*p*‐value
Recipients	2	34	841	877	0.022	2.5 (1.7–3.5)	<0.00001
Acute leukaemia	1	22	512	535	0.022	2.6 (1.7–4.0)	<0.0001
Lymphoproliferative disease	0	4	150	154	0.013	1.5 (0.6–4.1)	NS
Myeloproliferative disease	1	4	100	105	0.029	2.9 (1.2–7.1)	<0.05
Myelodysplastic syndrome	0	4	79	83	0.024	2.9 (1.1–8.0)	NS
Donors	1	16	652	669	0.013	1.5 (0.9–2.4)	NS
MFD	0	12	460	472	0.013	1.5 (0.8–2.7)	NS
MUD	1	4	192	197	0.015	1.5 (0.6–3.6)	NS
gnomAD v4.1.0 (Finnish)	1	547	31 473	32 021	0.008	‐	‐
*CHEK2* c.470T>C, p.Ile157Thr							
Recipients	5	77	795	877	0.050	1.9 (1.5–2.4)	<0.00001
Acute leukaemia	3	48	484	535	0.050	2.0 (1.5–2.6)	<0.0001
Lymphoproliferative disease	1	12	141	154	0.045	1.7 (1.0–3.0)	NS
Myeloproliferative disease	0	13	92	105	0.062	2.6 (1.5–4.7)	<0.01
Myelodysplastic syndrome	1	4	78	83	0.036	1.2 (0.5–3.0)	NS
Donors	2	45	622	669	0.037	1.4 (1.0–1.9)	<0.05
MFD	2	33	437	472	0.039	1.5 (1.1–2.1)	<0.05
MUD	0	12	185	197	0.030	1.2 (0.7–2.2)	NS
gnomAD v4.1.0 (Finnish)	19	1611	30 381	32 011	0.025	‐	‐

*Note*: Odds ratios (ORs) with 95% confidence intervals (CIs) are presented for each allele.

Abbreviations: Het, heterozygous; Hom, homozygous; MAF, minor allele frequency; MFD, matched family donor; MUD, matched unrelated donor; NS, not significant; OR, odds ratio, WT, wild type.

A further analysis was conducted on patients carrying either *CHEK2* c.1229del (*n* = 33) or c.470T>C (*n* = 79) variants (three patients carrying both were excluded from the analyses). We detected no difference in OS, relapse rate, NRM or GVHD either within the *CHEK2* variant groups or compared to patients without either variant (Figure 1C; Figures S2 and S3).

Both patients homozygous for the *CHEK2* c.1229del variant died of non‐relapse‐related causes within the first year after HSCT. Two of the five patients homozygous for the c.470C>T variant relapsed, leading to the death of one of them, while four survived until the end of the follow‐up period with a median follow‐up time of 5.2 years.

Seventeen (2.5%; 1 homozygous and 16 heterozygous) and 47 (7.0%; 2 homozygous and 45 heterozygous) of the 669 donors carried *CHEK2* variants c.1229del and c.470T>C respectively (Table S3). The prevalence of donors harbouring the *CHEK2* variants, some of whom were related to patients, exceeded that observed in the Finnish control population but fell below the prevalence noted in our patient cohort (Table 1). We did not find a significant impact on the recipient OS, relapse rate or NRM when comparing donors with the *CHEK2* variants to those with *CHEK2* wild type (Figure S4). In addition to *CHEK2* variants c.1229del and c.470T>C, we found three variants in the haematology panel in three donors and two variants in the oncology panel in six donors (Table S7). These variants had been identified in the respective HSCT recipients.

All three patients who received transplants from homozygous *CHEK2* carriers (one c.1229del and two c.470C>T) were alive at the end of follow‐up, with a median follow‐up time of 6.5 years with one experiencing relapse during that time.

Patients' or donors' *CHEK2* variants did not have a significant impact on the risk of death, relapse rate or non‐relapse mortality in these analyses (Figure S4). The results of the multivariate survival analysis were consistent with the acknowledged risk factors associated with HSCT outcomes (Table S8).

High‐quality care for patients with HM involves comprehensive genetic analyses and the integration of findings into clinical practice. Here, we focused on patients harbouring germline *CHEK2* variants who had undergone allogeneic HSCT due to HM. Our study consisted of 877 HSCT recipients including 118 with germline *CHEK2* variants. By comparing with population‐matched controls, we demonstrate that carriers of the *CHEK2* c.1229del or c.470T>C variants have a twofold increased risk of developing HMs requiring HSCT. These findings further support the role of *CHEK2* as one of the most prevalent inherited risk factors for HM.^11^, ^12^, ^13^

*CHEK2* carriership did not significantly affect 5‐year post‐HSCT outcomes, including overall survival, the incidence of GVHD or NRM. This contrasts with findings for other DDR genes, such as bi‐allelic mutations in Fanconi anaemia genes or *DDX41*, which have been associated with elevated risks of post‐transplant toxicity and acute GVHD.^5^, ^6^, ^7^ However, homozygosity for c.1229del may confer an increased risk of adverse post‐HSCT outcomes, which is shown by the early mortality observed within the first year following transplantation in both patients carrying the homozygous variant. Although short‐term outcomes following HSCT were favourable for heterozygous *CHEK2* carriers, these variants may still predispose individuals to an elevated risk of secondary malignancies with prolonged latency periods. Given that *CHEK2* is primarily associated with malignancies typically presenting in adulthood,^8^, ^9^, ^10^ prior findings emphasize the importance of long‐term post‐HSCT surveillance to assess the potential impact of *CHEK2* variants on late‐onset complications and secondary cancer development.

Importantly, although *CHEK2* variant carriership was associated with an increased predisposition to high‐risk HMs, a HSCT from a donor harbouring these variants, including homozygous carriers, did not negatively affect HSCT outcomes over the 5‐year follow‐up period. This suggests that stem cell donors harbouring *CHEK2* variants may have a comparatively smaller impact than high‐ or intermediate‐risk cancer predisposing gene variants such as *DDX41*, *GATA2* and *RUNX1*.^6^, ^15^ In conclusion, our findings exemplify the need for gene‐specific and possibly even variant‐specific studies aimed at the optimal integration of germline genetics into haematology clinical practice.

## AUTHOR CONTRIBUTIONS

These authors contributed equally: Atte K. Lahtinen and Maarja Karu. These authors contributed equally: Kirsi Jahnukainen, Outi Kilpivaara & Ulla Wartiovaara‐Kautto.

## FUNDING INFORMATION

This work was supported by the Research Council of Finland #349760, Cancer Foundation Finland, Sigrid Jusélius Foundation, the Finnish Special Governmental Subsidy for Health Sciences, Research, and Training, the Helsinki University Hospital Comprehensive Cancer Research Funding, the Finnish Funding Agency for Technology and Innovation (TEKES), the Väre Foundation for Pediatric Cancer Research, the iCAN Digital Precision Cancer Medicine, Cancer Foundation Finland, Ane and Signe Gyllenberg Foundation, foundation for Pediatric Research, the Swedish Childhood Cancer Foundation (KP2020‐0012) and the Birgitta and Carl‐Axel Rydbeck's Research Grant for Paediatric Research (2020‐00335, 2021‐00079 and 2023‐00380). We would also like to acknowledge support by the Finnish Medical Foundation, the Finnish Association of Hematology, the Ida Montin Foundation, the Biomedicum Helsinki Foundation and Jalmari and Rauha Ahokas Foundation.

## CONFLICT OF INTEREST STATEMENT

The authors declare no conflicts of interest.

## ETHICAL APPROVAL AND CONSENT TO PARTICIPATE

Samples and data were collected after obtaining informed consent from study participants or authorization by the ethics committee for deceased patients. The study has been approved by the Ethics Review Boards of each collaborating hospital and the Finnish National Supervisory Authority for Welfare and Health (Valvira). The permit numbers are #206/13/03/03/2016 (amendment 1Q/2023), #303/13/03/01/2011, HUS/114/2018, HUS/284/2019 and V/3235/2019 for the WES set and V/74832/2017, HUS/2152/2020 and ETMK 78/2012 for the SNP array set.

## Supporting information


Data S1.

## Data Availability

For original, de‐identified data, please contact the corresponding authors.

## References

[1] Baliakas P , Tesi B , Wartiovaara‐Kautto U , Stray‐Pedersen A , Friis LS , Dybedal I , et al. Nordic guidelines for germline predisposition to myeloid neoplasms in adults: recommendations for genetic diagnosis, clinical management and follow‐up. Hema. 2019;3(6):e321. 10.1097/HS9.0000000000000321 PMC6924562 31976490

[2] Clark A , Thomas S , Hamblin A , Talley P , Kulasekararaj A , Grinfeld J , et al. Management of patients with germline predisposition to haematological malignancies considered for allogeneic blood and marrow transplantation: best practice consensus guidelines from the UK Cancer Genetics Group (UKCGG), CanGene‐CanVar, NHS England Genomic Laboratory Hub (GLH) Haematological Malignancies Working Group and the British Society of Blood and Marrow Transplantation and cellular therapy (BSBMTCT). Br J Haematol. 2023;201(1):35–44. 36786081 10.1111/bjh.18682

[3] Godley LA , DiNardo CD , Bolton K . Germline predisposition in hematologic malignancies: testing, management, and implications. Am Soc Clin Oncol Educ Book. 2024;44(3):e432218. 38768412 10.1200/EDBK_432218

[4] Lahtinen AK , Koski J , Ritari J , Hyvärinen K , Koskela S , Partanen J , et al. Clinically relevant germline variants in allogeneic hematopoietic stem cell transplant recipients. Bone Marrow Transplant. 2023;58(1):39–45. 36195768 10.1038/s41409-022-01828-x PMC9812774

[5] Lum SH , Eikema DJ , Piepenbroek B , Wynn RF , Samarasinghe S , Dalissier A , et al. Outcome of hematopoietic stem cell transplantation in 813 pediatric patients with Fanconi anemia. Blood. 2024;144(12):1329–1342. 38968140 10.1182/blood.2023022751

[6] Saygin C , Roloff G , Hahn CN , Chhetri R , Gill S , Elmariah H , et al. Allogeneic hematopoietic stem cell transplant outcomes in adults with inherited myeloid malignancies. Blood Adv. 2023;7(4):549–554. 36001442 10.1182/bloodadvances.2022008172 PMC9979761

[7] Sakaguchi H , Yoshida N . Recent advances in hematopoietic cell transplantation for inherited bone marrow failure syndromes. Int J Hematol. 2022;116(1):16–27. 35633493 10.1007/s12185-022-03362-4

[8] Kilpivaara O , Vahteristo P , Falck J , Syrjäkoski K , Eerola H , Easton D , et al. CHEK2 variant I157T may be associated with increased breast cancer risk. Int J Cancer. 2004;111(4):543–547. 15239132 10.1002/ijc.20299

[9] Schreurs MAC , Schmidt MK , Hollestelle A , Schaapveld M , van Asperen CJ , Ausems MGEM , et al. Cancer risks for other sites in addition to breast in *CHEK2* c. 1100delC families. Genet Med. 2024;26(9):101171. 38828701 10.1016/j.gim.2024.101171

[10] Bychkovsky BL , Agaoglu NB , Horton C , Zhou J , Yussuf A , Hemyari P , et al. Differences in cancer phenotypes among frequent *CHEK2* variants and implications for clinical care—checking *CHEK2* . JAMA Oncol. 2022;8(11):1598–1606. 36136322 10.1001/jamaoncol.2022.4071 PMC9501803

[11] Freiman L , Larcher L , Tueur G , Vasquez N , Da Costa M , Duchmann M , et al. Germline *CHEK2* mutations in patients with myeloid neoplasms. Leukemia. 2024;38(4):908–911. 38378842 10.1038/s41375-024-02179-w

[12] Stubbins RJ , Korotev S , Godley LA . Germline *CHEK2* and *ATM* variants in myeloid and other hematopoietic malignancies. Curr Hematol Malig Rep. 2022;17(4):94–104. 35674998 10.1007/s11899-022-00663-7

[13] Koski JR , Langohr L , Räisänen T , Lahtinen AK , Hakkarainen M , Heckman CA , et al. Pathogenicity evaluation of coding germline variants identifies rare alleles enriched in hematological patients of a founder population. [Internet]. *medRxiv*. 2024 [cited 2024 Nov 1]. p. 2024. 10.23.24315723. Available from: https://www.medrxiv.org/content/10.1101/2024.10.23.24315723v1

[14] Chen S , Francioli LC , Goodrich JK , Collins RL , Kanai M , Wang Q , et al. A genomic mutational constraint map using variation in 76,156 human genomes. Nature. 2024;625(7993):92–100. 38057664 10.1038/s41586-023-06045-0 PMC11629659

[15] Williams LS , Williams KM , Gillis N , Bolton K , Damm F , Deuitch NT , et al. Donor‐derived malignancy and transplantation morbidity: risks of patient and donor genetics in allogeneic hematopoietic stem cell transplantation. Transplant Cell Ther. 2023;30:255–267. 37913908 10.1016/j.jtct.2023.10.018 PMC10947964

