# Supplementary material for: Impact of CHEK2 germline variants on haematological malignancy risk and outcomes of allogeneic HSCT
Source: Br J Haematol. 2025 Jun 12;207(2):636–41. doi: 10.1111/bjh.20196 (PMC12378959; doi:10.1111/bjh.20196)
Supplement: Supplementary file 1 — Data S1. [file BJH-207-636-s001.docx]

**Supplementary Data**

**Supplementary materials and methods………………………………………………...2**

**Supplementary tables……………………………………………………………………...3**

**Supplementary figures…………………………………………………………………...24**

**References………………………………………………………………………………….29**

**Supplementary materials and methods**

**Study set 1 (Whole exome sequencing data)**

The WES data set consisted of three patient cohorts: adult patient cohort from Helsinki University Hospital (HUH) and Turku University Hospital (Adult cohort 1), adult patients from the Finnish Bone Marrow Transplantation Registry (BMTR) (Adult cohort 2), and pediatric patients from HUH (Pediatric cohort). After excluding non-malignant hematological diseases and solid cancers, 391 patients remained in the WES data set (n = 138, Adult cohort 1; n = 130, Adult cohort 2; n = 123, Pediatric cohort).

The patients in the WES data set were first grouped according to the American College of Medical Genetics and Genomics (ACMG) classification having a pathogenic (P) or likely pathogenic (LP) variant, or no variants. We then grouped patients based on the presence of P/LP variants in a) genes predisposing to hereditary hematological malignancies, bone marrow failure syndromes or inborn errors of immunity (Hematology panel variant group), b) genes associated with predisposition to solid tumors, excluding *CHEK2* variant group (Oncology panel variant group), and c) *CHEK2* (*CHEK2* variant group). The control group consisted of patients that did not carry any P or LP predisposition variants in the genes listed in the panels, or *CHEK2* Ile157Thr. To focus on the effects of single gene variants, we excluded five patients because they carried multiple variants.

**Study set 2 (Single nucleotide polymorphism array data)**

To strengthen the analyses of the impact of *CHEK2* variants (c.1229del and c.470T>C), we used an independent HSCT cohort from the Finnish Bone Marrow Transplantation Registry. This set consisted of 486 patient-donor pairs with SNP array data (Adult cohort 3). We then excluded the 22 patients already included in the WES data set, 8 with non-malignant diseases and 48 patients diagnosed with lymphomas as an indication of HSCT.

The SNP array data was produced in the Finnish Institute of Molecular Medicine (FIMM) in Helsinki, Finland. Sample genotyping and imputation of the donor data has been described previously by Nihtilä *et al*.^1^

**HSCT** **Donors**

We also included the clinical and, when available, WES or SNP array data of the HSCT donors in our analyses. The WES information was available for 21 (15.2%) donors in Adult cohort 1, all donors in Adult cohort 2 (n = 130; 100%) and in 32 (26.0%) of the donors in the Pediatric cohort. We collected donors’ clinical information from the medical records gathered during the HSCT protocol. Germline data was available for 669 of 877 (76.3 %) donors, of which 507 had SNP arrays performed.

**Statistical analyses**

We conducted analyses of overall survival, relapse rate and non-relapse mortality, Kaplan-Meier, and log-rank tests. The covariates used in multivariate survival analyses and association testing were recipient and donor ages, donor type, graft, recipient and donor gender, preconditioning, diagnosis (acute leukemia or other), and transplantation year. Covariates included in multivariate analysis were chosen based on previous literature. *P* value <0.05 was regarded as statistically significant. Differences in minor allele frequencies between patients, donors, and the gnomAD Finnish population controls (version 4.1.0, n = 32 026) were compared by applying Fisher's exact tests.

**Supplementary Table 1.** Demographic data, transplant-related characteristics and *CHEK2* variant status by WES and SNP data of 877 recipients who underwent allogeneic hematopoietic stem cell transplantation.

| **Characteristic** | **Adult cohort 1** | **Adult cohort 2** | **Adult cohort 3** | **Pediatric cohort** |
| --- | --- | --- | --- | --- |
| **Number of patients** | **138** | **130** | **486** | **123** |
| Male, N (%) | 68 (49.3) | 65 (50.8) | 259 (53.3) | 80 (65.0) |
| Female, N (%) | 70 (50.7) | 63 (49.2) | 227 (46.7) | 43 (35.0) |
| NA | - | 2 | - | - |
| **Age at HSCT (years)** | **17 - 70** | **16 - 69** | **18 - 70** | **0 - 18** |
| Mean | 46.4 | 49.3 | 47.9 | 8.9 |
| Median | 48.3 | 52.1 | 50.1 | 8.3 |
|  | | | | |
| **Diagnosis, N (%)** |  | | | |
| **Acute leukemia** | **97 (70.3)** | **79 (60.8)** | **249 (51.2)** | **110 (89.4)** |
| Acute myeloid leukemia | 68 | 57 | 171 | 22 |
| Acute lymphoblastic leukemia | 29 | 22 | 78 | 88 |
| **Lymphoproliferative disease** | **12 (8.7)** | **30 (23.1)** | **112 (23.0)** | **0 (0)** |
| Multiple myeloma/Plasma cell leukemia | 12 | 21 | 89 | 0 |
| Chronic lymphocytic leukemia | 0 | 8 | 20 | 0 |
| Prolymphocytic leukemia | 0 | 1 | 3 | 0 |
| **Myeloproliferative neoplasms, N (%)** | **16 (11.6)** | **11 (8.5)** | **72 (14.8)** | **6 (4.9)** |
| Chronic myeloid leukemia | 8 | 3 | 49 | 6 |
| Myelofibrosis | 1 | 4 | 14 | 0 |
| Chronic myelomonocytic leukemia | 4 | 3 | 6 | 0 |
| MDS/MPN | 2 | 0 | 0 | 0 |
| MPN NOS | 1 | 0 | 3 | 0 |
| Mastocytosis | 0 | 1 | 0 | 0 |
| **Myelodysplastic neoplasms, N (%)** | **13 (9.4)** | **10 (7.7)** | **53 (10.9)** | **7 (5.7)** |
| Myelodysplastic syndrome | 13 | 10 | 53 | 4 |
| Juvenile myelomonocytic leukemia | 0 | 0 | 0 | 3 |
|  | | | | |
| **HSCT-related characteristics** |  | | | |
| Source of transplant, n (MUD/MFD/Haploidentical) | 109/25/4 | 0/138/0 | 168/318/0 | 69/51/3 |
| Type of transplant, n (PB/BM/PB+BM/CB/NA) | 117/20/0/1/0 | 100/26/0/0/4 | 283/201/2/0/0 | 0/105/18/0/0 |
| Conditioning, n (MAC/RIC/NA) | 116/22/0 | 92/36/2 | 386/100/0 | 122/1/0 |
|  |  |  |  |  |
| ***CHEK2* c.1229del, p. Cys410SerfsTer4** | | | | |
| Homozygous | 0 | 0 | 2 | 0 |
| Heterozygous | 4 | 3 | 22 | 5 |
| Wild-type | 134 | 127 | 462 | 118 |
| ***CHEK2* c.470T>C, p. Ile157Thr** | | | | |
| Homozygous | 0 | 1 | 3 | 1 |
| Heterozygous | 13 | 14 | 44 | 6 |
| Wild-type | 125 | 115 | 439 | 116 |

BM: bone marrow; CB: cord blood; HSCT: hematopoietic stem cell transplantation; MAC: myeloablative conditioning; MDS: myelodysplastic syndrome; MFD: matched family donor; MPN: myeloproliferative neoplasm; MUD: matched unrelated donor; NA: not available; NOS: not otherwise specified; PB: peripheral blood; RIC: reduced intensity conditioning; SNP: single nucleotide polymorphism; WES: whole exome sequencing.

**Supplementary Table 2. Gene panels.** 1A) Hematology gene panel containing genes predisposing to hereditary hematological malignancies, bone marrow failure syndromes or inborn errors of immunity and 1B) Oncology gene panel containing genes associated with predisposition to solid tumors.

Supplementary Table 1A

| **Gene** | **Inheritance** | **Syndrome** |
| --- | --- | --- |
| ABCD1 | XLR | Adrenoleukodystrophy, X-linked |
| ATRX | XLD | Alpha-thalassemia/mental retardation syndrome |
| SAMD9L | AD | Ataxia-pancytopenia syndrome, Monosomy 7 myelodysplasia and leukemia syndrome |
| ATM | AR | Ataxia-telangiectasia |
| CASP10 | AD | Autoimmune lymphoproliferative syndrome |
| CTLA4 | AD | Autoimmune lymphoproliferative syndrome |
| FAS | AD | Autoimmune lymphoproliferative syndrome |
| FASLG | AD | Autoimmune lymphoproliferative syndrome |
| PRKCD | AR | Autoimmune lymphoproliferative syndrome |
| TAZ | XLR | Barth syndrome |
| BLM | AR | Bloom syndrome |
| DNAJC21 | AR | Bone marrow failure syndrome |
| ERCC6L2 | AR | Bone marrow failure syndrome |
| SRP72 | AD | Bone marrow failure syndrome |
| MAP2K1 | AD | Cardiofaciocutaneous syndrome |
| MAP2K2 | AD | Cardiofaciocutaneous syndrome |
| RMRP | AR | Cartilage-hair hypoplasia |
| CTC1 | AR | Cerebroretinal microangiopathy with calcifications and cysts |
| STN1 (OBFC1) | AR | Cerebroretinal microangiopathy with calcifications and cysts |
| LYST | AR | Chediak-Higashi syndrome |
| CYBA | AR | Chronic granulomatous disease |
| CYBC1 (C17orf62) | AR | Chronic granulomatous disease |
| NCF1 | AR | Chronic granulomatous disease |
| NCF2 | AR | Chronic granulomatous disease |
| NCF4 | AR | Chronic granulomatous disease |
| CYBB | XLR | Chronic granulomatous disease, X-linked |
| MBD4 | AR | Clonal hematopoiesis and early-onset AML |
| CLPB | AR | CLPB deficiency |
| VPS13B | AR | Cohen syndrome |
| MTHFD1 | AR | Combined immunodeficiency and megaloblastic anemia with or without hyperhomocysteinemia |
| IKZF1 | AD | Common variable immunodeficiency |
| C15ORF41 | AR | Congenital dyserythropoietic anemia |
| CDAN1 | AR | Congenital dyserythropoietic anemia |
| KIF23 | AD | Congenital dyserythropoietic anemia |
| KLF1 | AD | Congenital dyserythropoietic anemia |
| SEC23B | AR | Congenital dyserythropoietic anemia |
| HRAS | AD | Costello syndrome |
| GATA1 | XLR | Diamond-Blackfan anemia |
| RPL11 | AD | Diamond-Blackfan anemia |
| RPL15 | AD | Diamond-Blackfan anemia |
| RPL18 | AD | Diamond-Blackfan anemia |
| RPL26 | AD | Diamond-Blackfan anemia |
| RPL27 | AD | Diamond-Blackfan anemia |
| RPL31 | AD | Diamond-Blackfan anemia |
| RPL35 | AD | Diamond-Blackfan anemia |
| RPL35A | AD | Diamond-Blackfan anemia |
| RPL36 | AD | Diamond-Blackfan anemia |
| RPL5 | AD | Diamond-Blackfan anemia |
| RPL9 | AD | Diamond-Blackfan anemia |
| RPS10 | AD | Diamond-Blackfan anemia |
| RPS15 | AD | Diamond-Blackfan anemia |
| RPS15A | AD | Diamond-Blackfan anemia |
| RPS17 | AD | Diamond-Blackfan anemia |
| RPS19 | AD | Diamond-Blackfan anemia |
| RPS24 | AD | Diamond-Blackfan anemia |
| RPS26 | AD | Diamond-Blackfan anemia |
| RPS27 | AD | Diamond-Blackfan anemia |
| RPS27A | AD | Diamond-Blackfan anemia |
| RPS28 | AD | Diamond-Blackfan anemia |
| RPS29 | AD | Diamond-Blackfan anemia |
| RPS7 | AD | Diamond-Blackfan anemia |
| TSR2 | XLR | Diamond-Blackfan anemia |
| ACD | AD, AR | Dyskeratosis congenita |
| DKC1 | XLR | Dyskeratosis congenita |
| NAF1 | AD | Dyskeratosis congenita |
| NHP2 | AR | Dyskeratosis congenita |
| NOP10 | AR | Dyskeratosis congenita |
| PARN | AD, AR | Dyskeratosis congenita |
| RTEL1 | AD, AR | Dyskeratosis congenita |
| TERC | AD | Dyskeratosis congenita |
| TERT | AD, AR | Dyskeratosis congenita |
| TINF2 | AD | Dyskeratosis congenita |
| WRAP53 | AR | Dyskeratosis congenita |
| CEBPA | AD | Familial AML |
| POT1 | AD | Familial chronic lymphocytic leukemia |
| PRF1 | AR | Familial hemophagocytic lymphohistiocytosis |
| DDX41 | AD | Familial myeloproliferative/lymphoproliferative neoplasms |
| RUNX1 | AD | Familial platelet disorder with associated myeloid malignancy |
| MPL | AD | Familial thrombocythemia |
| BRCA1 | AR | Fanconi anemia |
| BRCA2 | AR | Fanconi anemia |
| BRIP1 | AR | Fanconi anemia |
| ERCC4 | AR | Fanconi anemia |
| FANCA | AR | Fanconi anemia |
| FANCB | XLR | Fanconi anemia |
| FANCC | AR | Fanconi anemia |
| FANCD2 | AR | Fanconi anemia |
| FANCE | AR | Fanconi anemia |
| FANCF | AR | Fanconi anemia |
| FANCG | AR | Fanconi anemia |
| FANCI | AR | Fanconi anemia |
| FANCL | AR | Fanconi anemia |
| FANCM | AR | Fanconi anemia |
| MAD2L2 | AR | Fanconi anemia |
| PALB2 | AR | Fanconi anemia |
| RAD51 | AD | Fanconi anemia |
| RAD51C | AR | Fanconi anemia |
| SLX4 | AR | Fanconi anemia |
| UBE2T | AR | Fanconi anemia |
| XRCC2 | AR | Fanconi anemia |
| FLNA | AD | FNLA-related thrombocytopenia |
| GATA2 | AD | GATA2 deficiency |
| NBEAL2 | AR | Gray platelet syndrome |
| RAB27A | AR | Griscelli syndrome |
| STAT3 | AD | Hyper-IgE recurrent infection syndrome |
| MYD88 | AR | Immunodeficiency |
| TET2 | AR | Immunodeficiency |
| CD40LG | XLR | Immunodeficiency with hyper-IgM, X-linked |
| UNG | AR | Immunodeficiency with hyper IgM |
| AICDA | AR | Immunodeficiency with hyper-IgM |
| CD40 | AR | Immunodeficiency with hyper-IgM |
| TP53 | AD | Li-Fraumeni syndrome |
| LIG4 | AR | LIG4 syndrome |
| CD27 | AR | Lymphoproliferative syndrome |
| CD70 | AR | Lymphoproliferative syndrome |
| ITK | AR | Lymphoproliferative syndrome |
| SH2D1A | XLR | Lymphoproliferative syndrome, X-linked |
| XIAP | XLR | Lymphoproliferative syndrome, X-linked |
| TUBB1 | AD | Macrothrombocytopenia |
| MYH9 | AD | Macrothrombocytopenia and granulocyte inclusions with or without nephritis or sensorineural hearing loss |
| KIT | AD | Mastocytosis |
| MLH1 | AR | Mismatch repair cancer syndrome |
| MSH2 | AR | Mismatch repair cancer syndrome |
| MSH6 | AR | Mismatch repair cancer syndrome |
| PMS2 | AR | Mismatch repair cancer syndrome |
| SAMD9 | AD | Monosomy 7 myelodysplasia and leukemia syndrome, MIRAGE syndrome |
| MPO | AR | Myeloperoxidase deficiency |
| NF1 | AD | Neurofibromatosis |
| NBN | AR | Nijmegen breakage |
| LZTR1 | AD, AR | Noonan syndrome |
| NRAS | AD | Noonan syndrome |
| PTPN11 | AD | Noonan syndrome |
| RAF1 | AD | Noonan syndrome |
| RASA2 | AD | Noonan syndrome |
| RIT1 | AD | Noonan syndrome |
| RRAS | AD | Noonan syndrome |
| SOS1 | AD | Noonan syndrome |
| SOS2 | AD | Noonan syndrome |
| BRAF | AD | Noonan syndrome, Cardiofaciocutaneous syndrome |
| KRAS | AD | Noonan syndrome, Cardiofaciocutaneous syndrome, Autoimmune lymphoproliferative syndrome, type IV / RAS-associated autoimmune leukoproliferative disorder |
| CBL | AD | Noonan syndrome-like disorder |
| SHOC2 | AD | Noonan syndrome-like disorder |
| CA2 | AR | Osteopetrosis |
| CLCN7 | AD, AR | Osteopetrosis |
| LRP5 | AD | Osteopetrosis |
| OSTM1 | AR | Osteopetrosis |
| PLEKHM1 | AD, AR | Osteopetrosis |
| SNX10 | AR | Osteopetrosis |
| TCIRG1 | AR | Osteopetrosis |
| TNFRSF11A | AR | Osteopetrosis |
| TNFSF11 | AR | Osteopetrosis |
| USB1 | AR | Poikiloderma with neutropenia |
| HOXA11 | AD | Radioulnar synostosis with amegakaryocytic thrombocytopenia |
| MECOM | AD | Radioulnar synostosis with amegakaryocytic thrombocytopenia |
| RNF168 | AR | RIDDLE syndrome |
| CSF3R | AR | Severe congenital neutropenia |
| ELANE | AD | Severe congenital neutropenia |
| G6PC3 | AR | Severe congenital neutropenia |
| GFI1 | AD | Severe congenital neutropenia |
| HAX1 | AR | Severe congenital neutropenia |
| JAGN1 | AR | Severe congenital neutropenia |
| VPS45 | AR | Severe congenital neutropenia |
| SRP54 | AD | Severe congenital neutropenia, Shwachman-Diamond-like syndrome |
| ADA | AR | Severe combined immunodeficiency due to ADA deficiency |
| NHEJ1 | AR | Severe combined immunodeficiency with microcephaly, growth retardation, and sensitivity to ionizing radiation |
| DCLRE1C | AR | Severe combined immunodeficiency, Athabascan type |
| RAG1 | AR | Severe combined immunodeficiency, B cell-negative |
| RAG2 | AR | Severe combined immunodeficiency, B cell-negative |
| IL7R | AR | Severe combined immunodeficiency, T cell-negative, B-cell/natural killer-cell positive |
| PTPRC | AR | Severe combined immunodeficiency, T cell-negative, B-cell/natural killer-cell positive |
| JAK3 | AR | Severe combined immunodeficiency, T-negative/B-positive type |
| IL2RG | XLR | Severe combined immunodeficiency, X-linked |
| EFL1 (EFTUD1) | AR | Shwachman-Diamond syndrome |
| SBDS | AR | Shwachman-Diamond syndrome |
| PAX5 | AD | Susceptibility to ALL |
| JAK2 | AD | Thrombocythemia |
| THPO | AD | Thrombocythemia |
| ANKRD26 | AD | Thrombocytopenia |
| CYCS | AD | Thrombocytopenia |
| ETV6 | AD | Thrombocytopenia |
| IKZF5 | AD | Thrombocytopenia |
| PTPRJ | AR | Thrombocytopenia |
| SBF2 | AR | Thrombocytopenia, Charcot-Marie-Tooth disease |
| RBM8A | AR | Thrombocytopenia-absent radius syndrome |
| ADA2 (CECR1) | AR | Vasculitis, autoinflammation, immunodeficiency, and hematologic defects syndrome |
| EZH2 | AD | Weaver syndrome |
| CXCR4 | AD | WHIM syndrome |
| WIPF1 | AR | Wiskott-Aldrich syndrome |
| WAS | XLR | Wiskott-Aldrich syndrome, severe congenital neutropenia, thrombocytopenia |

Supplementary Table 1B

| **Gene** | **Inheritance** | **Cancer syndrome, cancer risk** |
| --- | --- | --- |
| AIP | AD | Pituitary adenoma |
| ALK | AD | Neuroblastoma |
| APC | AD | Familial adenomatous polyposis/colorectal cancer, other gastrointestinal cancers, CNS cancer |
| AR | AD | Prostate cancer, male breast cancer |
| ATM | AD | Breast cancer |
| ATR | AD | Oropharyngeal cancer, cutaneous telangiectasia and cancer syndrome, familial |
| AXIN2 | AD | Oligodontia-colorectal cancer syndrome |
| BAP1 | AD | Tumor predisposition syndrome (melanoma, malignant mesothelioma, lung adenocarcinoma, meningioma, and renal cell carcinoma) |
| BARD1 | AD | Breast cancer |
| BLM | AR | Bloom syndrome (squamous cell skin cancer, leukemia, lymphoma, and gastrointestinal tract cancer) |
| BMPR1A | AD | Juvenile polyposis syndrome (stomach, small intestine, colon, and rectum cancer) |
| BRAF | AD | LEOPARD syndrome, Noonan syndrome, Cardiofaciocutaneous syndrome |
| BRCA1 | AD | Breast cancer, ovarian cancer, pancreatic cancer |
| BRCA2 | AD | Breast cancer, ovarian cancer, pancreatic cancer, medulloblastoma, prostate cancer, Wilms tumor |
| BRIP1 | AD | Breast cancer |
| BUB1B | AR | Mosaic variegated aneuploidy syndrome |
| CBL | AD | Noonan syndrome-like disorder |
| CDC73 | AD | Parathyroid carcinoma, hyperparathyroidism-jaw tumor syndrome |
| CDH1 | AD | Gastric cancer, breast cancer, prostate cancer |
| CDK4 | AD | Malignant melanoma, familial |
| CDKN1B | AD | Multiple endocrine neoplasia |
| CDKN1C | AD | Beckwith-Wiedemann syndrome |
| CDKN2A | AD | Pancreatic cancer, familial malignant melanoma |
| CEP57 | AR | Mosaic variegated aneuploidy syndrome |
| CHEK2 | AD | Breast cancer, colorectal cancer |
| CYLD | AD | Brooke-Spiegler syndrome, familial cylindromatosis, multiple familial trichoepithelioma |
| DDB2 | AR | Xeroderma pigmentosum |
| DICER1 | AD | DICER1 syndrome (pleuropulmonary blastoma, Sertoli-Leydig cell tumor, tyhroid cancer) |
| DIS3L2 | AR | Perlman syndrome |
| EGFR | AD | Familial lung cancer |
| EPCAM | AD | Colorectal cancer, endometrial cancer |
| ERCC2 | AR | Xeroderma pigmentosum |
| ERCC3 | AR | Xeroderma pigmentosum |
| ERCC4 | AR | Xeroderma pigmentosum |
| ERCC5 | AR | Xeroderma pigmentosum |
| EXT1 | AD | Multiple exostoses |
| EXT2 | AD | Multiple exostoses |
| FAM111B | AD | Pancreatic cancer (POIKTMP - Poikiloderma, hereditary fibrosing, with tendon contractures, myopathy, and pulmonary fibrosis) |
| FANCM | AD | Breast cancer |
| FH | AD | Hereditary leiomyomatosis and renal cell cancer |
| FLCN | AD | Birt-Hogg-Dube syndrome |
| GALNT12 | AD | Colorectal cancer |
| GPC3 | XLR | Simpson Golabi Behmel syndrome |
| GREM1 | AD | Hereditary Mixed Polyposis Syndrome |
| HNF1A | AR | Hepatocellular carcinoma, hepatic adenoma, renal cell carcinoma |
| HOXB13 | AD | Familial prostate cancer |
| HRAS | AD | Costello syndrome |
| KIT | AD | Gastrointestinal stromal tumor |
| KRAS | AD | Noonan syndrome, Cardiofaciocutaneous syndrome |
| LZTR1 | AD, AR | Noonan syndrome, schwannomatosis |
| MAP2K1 | AD | Cardiofaciocutaneous syndrome |
| MAP2K2 | AD | Cardiofaciocutaneous syndrome |
| MAX | AD | Pheochromocytoma |
| MEN1 | AD | Multiple endocrine neoplasia |
| MET | AD | Hereditary papillary renal carcinoma |
| MITF | AD | Renal cancer, melanoma |
| MLH1 | AD; AR | Muir-Torre syndrome, hereditary nonpolyposis colorectal cancer; Mismatch repair cancer syndrome |
| MLH3 | AD | Hereditary nonpolyposis colorectal cancer, endometrial cancer |
| MSH2 | AD; AR | Muir-Torre syndrome, hereditary nonpolyposis colorectal cancer; Mismatch repair cancer syndrome |
| MSH3 | AR | Familial adenomatous polyposis |
| MSH6 | AD; AR | Hereditary nonpolyposis colorectal cancer, endometrial cancer; Mismatch repair cancer syndrome |
| MUTYH | AR | Familial adenomatous polyposis (MUTYH-associated polyposis syndrome) |
| NBN | AR | Nijmegen breakage syndrome |
| NF1 | AD | Neurofibromatosis |
| NF2 | AD | Neurofibromatosis |
| NRAS | AD | Noonan syndrome |
| NTHL1 | AR | Familial adenomatous polyposis |
| PALB2 | AD | Breast cancer, pancreatic cancer, gastrointestinal cancer |
| PDGFRA | AD | Familial gastrointestinal stromal tumour |
| PHOX2B | AD | Neuroblastoma |
| PMS2 | AD; AR | Hereditary nonpolyposis colorectal cancer; Mismatch repair cancer syndrome |
| POLD1 | AD | Colorectal cancer |
| POLE | AD | Colorectal cancer |
| POLH | AR | Xeroderma pigmentosum |
| POT1 | AD | Malignant melanoma, glioma |
| PRKAR1A | AD | Carney complex |
| PTCH1 | AD | Basal cell nevus syndrome |
| PTEN | AD | Cowden syndrome |
| PTPN11 | AD | Noonan syndrome |
| RAD51C | AD | Breast-ovarian cancer |
| RAD51D | AD | Breast-ovarian cancer |
| RAF1 | AD | Noonan syndrome |
| RASA2 | AD | Noonan syndrome |
| RB1 | AD | Retinoblastoma |
| RECQL4 | AR | Rothmund-Thomson syndrome |
| REST | AD | Wilms tumor |
| RET | AD | Multiple endocrine neoplasia |
| RHBDF2 | AD | Tylosis with esophageal cancer |
| RIT1 | AD | Noonan syndrome |
| RPS20 | AD | Colorectal cancer |
| RRAS | AD | Noonan-like syndrome |
| SDHA | AD | Paraganglioma, pheochromocytoma |
| SDHAF2 | AD | Paraganglioma, pheochromocytoma |
| SDHB | AD | Paraganglioma, pheochromocytoma, gastrointestinal stromal tumor |
| SDHC | AD | Paraganglioma, pheochromocytoma, gastrointestinal stromal tumor |
| SDHD | AD | Paraganglioma, pheochromocytoma, gastrointestinal stromal tumor |
| SHOC2 | AD | Noonan-like syndrome |
| SMAD4 | AD | Juvenile polyposis/hereditary hemorrhagic telangiectasia syndrome |
| SMARCA4 | AD | Rhabdoid tumor predisposition syndrome |
| SMARCB1 | AD | Rhabdoid tumor predisposition syndrome, schwannomatosis |
| SOS1 | AD | Noonan syndrome |
| SOS2 | AD | Noonan syndrome |
| STK11 | AD | Peutz-Jeghers syndrome |
| SUFU | AD | Basal cell nevus syndrome, medulloblastoma |
| TERT | AD | Malignant melanoma |
| TMEM127 | AD | Pheochromocytoma, paraganglioma |
| TP53 | AD | Li-Fraumeni syndrome |
| TSC1 | AD | Tuberous sclerosis |
| TSC2 | AD | Tuberous sclerosis |
| VHL | AD | Von Hippel-Lindau syndrome |
| WRN | AR | Werner syndrome |
| WT1 | AD | Wilms tumor |
| XPA | AR | Xeroderma pigmentosum |
| XPC | AR | Xeroderma pigmentosum |

ALL: acute lymphoblastic leukemia; AML: acute myeloid leukemia; AD: autosomal dominant; AR: autosomal recessive; XLD: X-linked dominant; XLR: X-linked recessive.

**Supplementary Table 3.** Demographic data and *CHEK2* variant status by WES and SNP data of the 669 HSCT donors.

| **Characteristic** | **Adult cohort 1** | **Adult cohort 2** | **Adult cohort 3** | **Pediatric cohort** |
| --- | --- | --- | --- | --- |
| **Number of donors** | **138** | **130** | **486** | **123** |
| **Gender, N (%)** | **137 (99)** | **130 (100)** | **471 (97)** | **122 (99)** |
| Male, N (%) | 91 (66.4) | 74 (56.9) | 276 (58.6) | 83 (68.0) |
| Female, N (%) | 46 (33.6) | 56 (43.1) | 195 (41.4) | 39 (32.0) |
| **Donor age at harvest, years (%)** | **19-64 (99)** | **12-71 (98)** | **19-72 (56)** | **0-53 (77)** |
| Mean | 37.0 | 47.8 | 40.1 | 23.0 |
| Median | 36.0 | 49.2 | 39.0 | 23.0 |
| **Donor type (MFD/MUD)** | 29/109 | 130/0 | 318/160 | 54/69 |
|  | | | | |
| **Genetic testing data available, N (%)** | **21 (15.2)** | **130 (100)** | **486 (100)** | **32 (26.0)** |
| WES, N | 0 | 130 | 0 | 32 |
| SNP array, N | 21 | 0 | 486 | 0 |
| **Gender data available, N (%)** | **21 (100)** | **130 (100)** | **471 (97)** | **32 (100)** |
| Male, N (%) | 12 (57.1) | 74 (56.9) | 276 (58.6) | 21 (65.6) |
| Female, N (%) | 9 (42.9) | 56 (43.1) | 195 (41.4) | 11 (34.4) |
| **Donor age range at HSCT, years (%)** | **24-55 (100)** | **12-71 (98)** | **19-72 (56)** | **2-51 (75)** |
| Mean | 38.5 | 47.8 | 40.1 | 20.9 |
| Median | 35.9 | 49.2 | 39.0 | 16.5 |
| **Donor type (MFD/MUD)** | 4/17 | 130/0 | 318/160 | 20/12 |
|  | | | | |
| ***CHEK2* c.1229del, p. Cys410SerfsTer4** | | | | |
| Homozygous | 0 | 0 | 1 | 0 |
| Heterozygous | 0 | 2 | 14 | 0 |
| Wild-type | 21 | 128 | 471 | 32 |
| ***CHEK2* c.470T>C,** **p. Ile157Thr** | | | | |
| Homozygous | 1 | 1 | 0 | 0 |
| Heterozygous | 3 | 11 | 30 | 1 |
| Wild-type | 17 | 118 | 456 | 31 |

HSCT: hematopoietic stem cell transplantation; MFD: matched family donor; MUD: matched unrelated donor; SNP: single nucleotide polymorphism; WES: whole exome sequencing.

**Supplementary Table 4.** Pathogenic or likely pathogenic gene variants and *CHEK2* c.470T>C variants identified in recipients in Adult cohort 1, Adult cohort 2 and Pediatric cohort grouped according to the American College of Medical Genetics and Genomics (ACMG) classification. Five patients with multiple variants not included.

| **Patient** | **Diagnosis** | **Age at HSCT** | **Donor Type** | **Gene  (Transcript)** | **Base change,  Amino acid change** | **Genotype** | **VAF** | **Clinvar** | **InterVar (Criteria)** | **ACMG classification**^‡^ | **AF (Fin)** | **AF (All)** |
| --- | --- | --- | --- | --- | --- | --- | --- | --- | --- | --- | --- | --- |
| **CHEK2 variant group (Adult cohort 1)** | | | | | | | | | | | | |
| A004 | AML | 63 | MFD | CHEK2  (NM_007194.4) | c.470T>C, p.Ile157Thr | Het(28/58) | 0.48 | - | - | VUS | 0.02473 | 0.00403 |
| A005 | AML | 57 | MUD | CHEK2  (NM_007194.4) | c.470T>C, p.Ile157Thr | Het(138/275) | 0.50 | - | - | VUS | 0.02473 | 0.00403 |
| A007 | CML | 56 | MFD | CHEK2  (NM_007194.4) | c.470T>C, p.Ile157Thr | Het(24/53) | 0.45 | - | - | VUS | 0.02473 | 0.00403 |
| A045 | AML | 59 | MMFD | CHEK2  (NM_007194.4) | c.1229del, p. Cys410SerfsTer4 | Het(8/16) | 0.50 | CIP | VUS (PSV1) | Pathogenic (PVS1 VS, PP5 St, PM2 M, PP3 Su) | 0.00874 | 0.00205 |
| A049 | tAML | 55 | MUD | CHEK2  (NM_007194.4) | c.470T>C, p.Ile157Thr | Het(63/128) | 0.49 | - | - | VUS | 0.02473 | 0.00403 |
| A053 | tAML | 70 | MUD | CHEK2  (NM_007194.4) | c.1229del, p. Cys410SerfsTer4 | Het(20/30) | 0.67 | CIP | VUS (PSV1) | Pathogenic (PVS1 VS, PP5 St, PM2 M, PP3 Su) | 0.00874 | 0.00205 |
| A067 | MM | 49 | MUD | CHEK2  (NM_007194.4) | c.470T>C, p.Ile157Thr | Het(18/37) | 0.49 | - | - | VUS | 0.02473 | 0.00403 |
| A073 | CML | 36 | MUD | CHEK2  (NM_007194.4) | c.470T>C, p.Ile157Thr | Het(39/94) | 0.41 | - | - | VUS | 0.02473 | 0.00403 |
| A076 | T-ALL | 39 | MUD | CHEK2  (NM_007194.4) | c.470T>C, p.Ile157Thr | Het(61/121) | 0.50 | - | - | VUS | 0.02473 | 0.00403 |
| A082 | ALL | 33 | MUD | CHEK2  (NM_007194.4) | c.1229del, p. Cys410SerfsTer4 | Het(24/56) | 0.43 | CIP | VUS (PSV1) | Pathogenic (PVS1 VS, PP5 St, PM2 M, PP3 Su) | 0.00874 | 0,00205 |
| A096 | ALL | 44 | MUD | CHEK2  (NM_007194.4) | c.470T>C, p.Ile157Thr | Het(39/69) | 0.57 | - | - | VUS | 0.02473 | 0.00403 |
| A109 | ALL | 24 | MUD | CHEK2  (NM_007194.4) | c.470T>C, p.Ile157Thr | Het(21/52) | 0.40 | - | - | VUS | 0.02473 | 0.00403 |
| A117 | AML | 39 | MUD | CHEK2  (NM_007194.4) | c.470T>C, p.Ile157Thr | Het(55/109) | 0.50 | - | - | VUS | 0.02473 | 0.00403 |
| A130 | AML | 45 | MUD | CHEK2  (NM_007194.4) | c.1229del, p. Cys410SerfsTer4 | Het(16/29) | 0.55 | CIP | VUS (PSV1) | Pathogenic (PVS1 VS, PP5 St, PM2 M, PP3 Su) | 0.00874 | 0.00205 |
| A135 | ALL | 36 | MFD | CHEK2  (NM_007194.4) | c.470T>C, p.Ile157Thr | Het(31/83) | 0.37 | - | - | VUS | 0.02473 | 0.00403 |
| A136 | AML | 29 | MUD | CHEK2  (NM_007194.4) | c.470T>C, p.Ile157Thr | Het(23/44) | 0.52 | - | - | VUS | 0.02473 | 0.00403 |
| **CHEK2 variant group (Adult cohort 2)** | | | | | | | | | | | | |
| V033 | MM | 53 | NA | CHEK2  (NM_007194.4) | c.470T>C, p.Ile157Thr | Het(32/72) | 0.44 | - | - | VUS | 0.02473 | 0.00403 |
| V038 | AML | 60 | NA | CHEK2  (NM_007194.4) | c.1229del, p. Cys410SerfsTer4 | Het(4/12) | 0.33 | CIP | VUS (PVS1) | Pathogenic (PVS1 VS, PP5 St, PM2 M, PP3 Su) | 0.00874 | 0.00205 |
| V044 | ALL | 18 | NA | CHEK2  (NM_007194.4) | c.1229del, p. Cys410SerfsTer4 | Het(5/10) | 0.50 | CIP | VUS (PVS1) | Pathogenic (PVS1 VS, PP5 St, PM2 M, PP3 Su) | 0.00874 | 0.00205 |
| V052 | MM | 43 | NA | CHEK2  (NM_007194.4) | c.470T>C, p.Ile157Thr | Het(54/113) | 0.48 | - | - | VUS | 0.02473 | 0.00403 |
| V059 | MPAL | 38 | NA | CHEK2  (NM_007194.4) | c.470T>C, p.Ile157Thr | Het(32/60) | 0.53 | - | - | VUS | 0.02473 | 0.00403 |
| V060 | AML | 48 | NA | CHEK2  (NM_007194.4) | c.470T>C, p.Ile157Thr | Het(28/55) | 0.51 | - | - | VUS | 0.02473 | 0.00403 |
| V062 | MDS | 59 | NA | CHEK2  (NM_007194.4) | c.470T>C, p.Ile157Thr | Het(30/65) | 0.46 | - | - | VUS | 0.02473 | 0.00403 |
| V063 | AML | 41 | NA | CHEK2  (NM_007194.4) | c.1229del, p. Cys410SerfsTer4 | Het(10/12) | 0.83 | CIP | VUS (PVS1) | Pathogenic (PVS1 VS, PP5 St, PM2 M, PP3 Su) | 0.00874 | 0.00205 |
| V065 | ALL | 41 | NA | CHEK2  (NM_007194.4) | c.470T>C, p.Ile157Thr | Het(49/114) | 0.43 | - | - | VUS | 0.02473 | 0.00403 |
| V067 | AML | 55 | NA | CHEK2  (NM_007194.4) | c.470T>C, p.Ile157Thr | Het(35/63) | 0.56 | - | - | VUS | 0.02473 | 0.00403 |
| V069 | CLL | 52 | NA | CHEK2  (NM_007194.4) | c.319+2T>A, splice cite variant | Het(22/40) | 0.55 | P/LP | P (PVS1, PM2, PP3, PP5) | Pathogenic (PVS1 VS, PP5 St, PM2 M, PP3 Su) | 0.00056 | 0.00007 |
| V073 | MF | 62 | NA | CHEK2  (NM_007194.4) | c.470T>C, p.Ile157Thr | Het(29/65) | 0.45 | - | - | VUS | 0.02473 | 0.00403 |
| V077 | MM | 56 | NA | CHEK2  (NM_007194.4) | c.538C>T, p.Arg180Cys | Het(31/60) | 0.52 | - | - | VUS | 0.00032 | 0.00103 |
| V078 | ALL | 31 | NA | CHEK2  (NM_007194.4) | c.470T>C, p.Ile157Thr | Het(33/63) | 0.52 | - | - | VUS | 0.02473 | 0.00403 |
| V0103 | ALL | 59 | NA | CHEK2  (NM_007194.4) | c.470T>C, p.Ile157Thr | Het(21/30) | 0.7 | - | - | VUS | 0.02473 | 0.00403 |
| V110 | AML | 64 | NA | CHEK2  (NM_007194.4) | c.470T>C, p.Ile157Thr | Het(37/71) | 0.52 | - | - | VUS | 0.02473 | 0.00403 |
| V122 | AML | 24 | NA | CHEK2  (NM_007194.4) | c.470T>C, p.Ile157Thr | Hom(63/63) | 1.0 | - | - | VUS | 0.02473 | 0.00403 |
| V129 | AML | 29 | NA | CHEK2  (NM_007194.4) | c.470T>C, p.Ile157Thr | Het(36/68) | 0.53 | - | - | VUS | 0.02473 | 0.00403 |
| **CHEK2 variant group (Pediatric cohort)** | | | | | | | | | | | | |
| P066 | ALL | 16 | MUD | CHEK2  (NM_007194.4) | c.1229del, p. Cys410SerfsTer4 | Het(66/133) | 0.50 | CIP | VUS (PSV1) | Pathogenic (PVS1 VS, PP5 St, PM2 M, PP3 Su) | 0.00874 | 0.00205 |
| P092 | ALL | 16 | MUD | CHEK2  (NM_007194.4) | c.1229del, p. Cys410SerfsTer4 | Het(53/113) | 0.47 | CIP | VUS (PSV1) | Pathogenic (PVS1 VS, PP5 St, PM2 M, PP3 Su) | 0.00874 | 0.00205 |
| P126 | ALL | 14 | MFD | CHEK2  (NM_007194.4) | c.470T>C, p.Ile157Thr | Het(45/91) | 0.49 | - | - | VUS | 0.02473 | 0.00403 |
| P163 | ALL | 10 | MFD | CHEK2  (NM_007194.4) | c.470T>C, p.Ile157Thr | Het(64/120) | 0.53 | - | - | VUS | 0.02473 | 0.00403 |
| P188 | ALL | 15 | MFD | CHEK2  (NM_007194.4) | c.1229del, p. Cys410SerfsTer4 | Het(51/121) | 0.42 | CIP | VUS (PSV1) | Pathogenic (PVS1 VS, PP5 St, PM2 M, PP3 Su) | 0.00874 | 0.00205 |
| P205 | ALL | 3 | MUD | CHEK2  (NM_007194.4) | c.470T>C, p.Ile157Thr | Het(107/237) | 0.45 | - | - | VUS | 0.02473 | 0.00403 |
| P245 | ALL | 5 | MFD | CHEK2  (NM_007194.4) | c.470T>C, p.Ile157Thr | Het(75/170) | 0.44 | - | - | VUS | 0.02473 | 0.00403 |
| P253 | ALL | 4 | MUD | CHEK2  (NM_007194.4) | c.470T>C, p.Ile157Thr | Hom(231/231) | 1.0 | - | - | VUS | 0.02473 | 0.00403 |
| P295 | ALL | 10 | MUD | CHEK2  (NM_007194.4) | c.1229del, p. Cys410SerfsTer4 | Het(53/110) | ###### | CIP | VUS (PSV1) | Pathogenic (PVS1 VS, PP5 St, PM2 M, PP3 Su) | 0.00874 | 0.00205 |
| P317 | ALL | 6 | MMUD | CHEK2  (NM_007194.4) | c.470T>C, p.Ile157Thr | Het(66/140) | 0.47 | - | - | VUS | 0.02473 | 0.00403 |
| **Hematology gene variant group (Adult cohort 1)** | | | | | | | | | | | | |
| A038 | AML | 64 | MMUD | DDX41  (NM_016222.4) | c.3G>A, p.Met1Ile | Het(18/45) | 0.40 | P/LP | VUS (PP3, PP5) | P (PVS1 VS, PP5 St, PP3 Su) | 0.00005 | 0.00009 |
| A039 | ALL | 55 | MFD | RUNX1 (NM_001754.5) | c.611G>A, p.Arg204Gln | Het(32/70) | 0.46 | P | LP (PM1, PM2, PP3, PP5) | P (PP5 VS, PP3 St, PM1 M, PM2 M, PM5 M, PP2 Su) | NA | NA |
| A041 | MDS/MPN | 59 | MUD | TERT (NM_198253.3) | c.2320C>T, p.Arg774Ter | Het(59/114) | 0.52 | P | P (PVS1, PM2, PP5) | P (PVS1 VS, PM2 M, PP5 M, BP4 Su) | 0.00052 | 0.00006 |
| A042 | MM | 56 | MUD | POT1 (NM_015450.3) | c.547-1G>A, splice cite variant | Het(31/51) | 0.61 | UNK | P (PVS1, PM2, PP3) | P (PVS1 VS, PM2 M, PP3 Su) | NA | NA |
| A063 | AML | 38 | MUD | ERCC6L2 (ENST00000288985.7) | c.1457del, p.Ile486ThrfsTer36 | Hom(97/97) | 1.00 | UNK | VUS (PVS1) | P (PVS1 VS, PP5 St, PM2 M, PP3 Su) | 0.00511 | 0.00048 |
| A095^†^ | MDS | 41 | MUD | GATA2 (NM_032638.5) | c.1061C>T, p.Thr354Met | Het(43/99) | 0.43 | P | LP (PM1, PM2, PP3, PP5) | P (PP5 VS, PM1 M, PM2 M, PM5 M, PP2 Su, PP3 Su) | NA | NA |
| A102^†^ | MDS | 32 | MMUD | GATA2 (NM_032638.5) | c.982C>T, p.Gln328Ter | Het(38/79) | 0.48 | UNK | P (PVS1, PM2, PP3) | P (PVS1 VS, PM2 M, PP3 Su) | NA | NA |
| **Hematology gene variant group (Adult cohort 2)** | | | | | | | | | | | | |
| V009 | AML | 44 | NA | GATA2 (NM_032638.5) | c.1061C>T, p.Thr354Met | Het(5/15) | 0.33 | P | LP (PM1, PM2, PP3, PP5) | P (PP5 VS, PM1 M, PM2 M, PM5 M, PP2 Su, PP3 Su) | NA | NA |
| V092 | AML | 63 | NA | SAMD9 (NM_001193307.1) | c.1030C>T, p.Arg344Ter | Het(19/38) | 0.50 | P | VUS (PM2, PP5) | P (PVS1 VS, PM2 M, PP5 Su, BP4 Su) | NA | 0.00004 |
| V102 | AML | 36 | NA | ANKRD26 (NM_014915.3) | c.2087_2088del, p.Glu696GlyfsTer3 | Het(10/22) | 0.45 | UNK | LP (PVS1, PM2) | P (PVS1 VS, PM2 M, PP3 Su) | NA | NA |
| V117 | ALL | 48 | NA | ANKRD26 (NM_014915.3) | c.4633G>T, p.Glu1545Ter | Het(24/40) | 0.60 | UNK | LP (PVS1, PM2) | P (PVS1 VS, PM2 M, PP3 Su) | NA | 0.00000 |
| **Hematology gene variant group (Pediatric cohort)** | | | | | | | | | | | | |
| P169 | ALL | 5 | MUD | TP53 (ENST00000269305.4) | c.535C>T, p.His179Tyr | Het(215/255) | 0.84 | P/LP | LP (PM1, PM2, PM5, PP3, PP5) | P (PP5 VS, PM1 St, PM2 M, PM5 M, PP2 Su, PP3 Su) | NA | NA |
| P151 | ALL | 14 | MUD | TP53 (ENST00000269305.4) | c.733G>A, p.Gly245Ser | Het(77/183) | 0.42 | P | LP (PM1, PM2, PM5, PP3, PP5) | P (PP5 VS, PM1 St, PM2 M, PM5 M, PP2 Su, PP3 Su) | NA | NA |
| P184^†^ | AML | 12 | MUD | GATA2 (NM_032638.5) | c.1061C>T, p.Thr354Met | Het(139/279) | 0.50 | P | LP (PM1, PM2, PP3, PP5) | P (PP5 VS, PM1 M, PM2 M, PM5 M, PP2 Su, PP3 Su) | NA | NA |
| **Oncology gene variant group (Adult cohort 1)** | | | | | | | | | | | | |
| A003 | CMML | 66 | MUD | FANCM (NM_020937.4) | c.5101C>T, p.Gln1701Ter | Het(60/129) | 0.47 | P/LP | VUS (PVS1, PP5) | P (PVS1 VS, PP5 VS, PM2 M, BP4 Su) | 0.00823 | 0.00129 |
| A047 | AML | 57 | MUD | ATR (NM_001184.4) | c.6463G>T, p.Glu2155Ter | Het(50/117) | 0.43 | UNK | P (PVS1, PM2, PP3) | P (PVS1 VS, PM2 M, PP3 Su) | NA | NA |
| A069 | sAML | 38 | MFD | HOXB13 (NM_006361.6) | c.251G>A, p.Gly84Glu | Het(30/42) | 0.71 | P/LP | VUS (PM1, PP2, PP3, PP5) | VUS (PP5 St, PP3 Su) | 0.00783 | 0.00186 |
| A084 | ALL | 48 | MUD | HOXB13 (NM_006361.6) | c.251G>A, p.Gly84Glu | Het(33/60) | 0.55 | P/LP | VUS (PM1, PP2, PP3, PP5) | VUS (PP5 St, PP3 Su) | 0.00783 | 0.00186 |
| A089 | AML | 45 | MUD | FANCM (NM_020937.4) | c.5791C>T, p.Arg1931Ter | Het(104/248) | 0.42 | P | P (PVS1, PP3, PP5) | P (PVS1 VS, PM2 M, PP3 Su) | 0.00448 | 0.00101 |
| A094 | AML | 46 | MUD | AR (NM_000044.6) | c.2180G>T, p.Arg727Leu | Hom(48/48) | 1.00 | P | VUS (PM1, PP3, PP5) | LP (PM1 M, PP2 Su, PP3 Su, PP5 Su, BS2 St) | 0.00835 | NA |
| A123 | ALL | 41 | MUD | BRCA1 (NM_007300.4) | c.4097-2A>G, splice cite variant | Het(14/24) | 0.58 | P | P (PVS1, PM2, PP3, PP5) | P (PVS1 VS, PP5 VS, PM2 M, PP3 Su) | NA | NA |
| A125 | AML | 47 | MFD | FANCM (NM_020937.4) | c.1491dup, p.Gln498ThrfsTer7 | Het(75/149) | 0.50 | P/LP | VUS (PVS1, PP5) | P (PVS1 VS, PP5 VS, PM2 M, PP3 Su) | 0.00023 | 0.00004 |
| **Oncology gene variant group (Adult cohort 2)** | | | | | | | | | | | | |
| V053 | AML | 53 | NA | HOXB13 (NM_006361.6) | c.251G>A, p.Gly84Glu | Het(5/10) | 0.50 | P/LP | VUS (PM1,PP2, PP3, PP5) | VUS (PP5 St, PP3 Su) | 0.00783 | 0.00186 |
| V081 | AML | 28 | NA | FANCM (NM_020937.4) | c.5101C>T, p.Gln1701Ter | Het(24/41) | 0.59 | P/LP | VUS (PVS1, PP5) | P (PVS1 VS, PP5 VS, PM2 M, BP4 Su) | 0.00823 | 0.00129 |
| V093 | AML | 59 | NA | FANCM (NM_020937.4) | c.5101C>T, p.Gln1701Ter | Het(24/49) | 0.49 | P/LP | VUS (PVS1, PP5) | P (PVS1 VS, PP5 VS, PM2 M, BP4 Su) | 0.00823 | 0.00129 |
| V114 | PCL | 50 | NA | FANCM (NM_020937.4) | c.5101C>T, p.Gln1701Ter | Het(24/40) | 0.60 | P/LP | VUS (PVS1, PP5) | P (PVS1 VS, PP5 VS, PM2 M, BP4 Su) | 0.00823 | 0.00129 |
| V119 | CLL | 61 | NA | ATM (NM_000051.4) | c.6198+1G>A, splice cite variant | Het(14/28) | 0.50 | P/LP | P (PVS1, PM2, PP3, PP5) | P (PVS1 VS, PP5 St, PM2 M, PP3 Su) | NA | 0.00000 |
| V130 | MDS | 54 | NA | RAD51D (NM_001142571.2) | c.636+1G>A, splice cite variant | Het(7/9) | 0.78 | P/LP | P (PVS1, PP3, PP5) | P (PVS1 VS, PP5 St, PM2 M, PP3 Su) | NA | 0.00000 |
| **Oncology gene variant group (Pediatric cohort)** | | | | | | | | | | | | |
| P022 | ALL | 10 | MFD | FANCM (NM_020937.4) | c.5791C>T, p.Arg1931Ter | Het(67/120) | 0.56 | P | P (PVS1, PP3, PP5) | P (PVS1 VS, PM2 M, PP3 Su) | 0.00448 | 0.00101 |
| P042 | CML | 14 | MFD | FANCM (NM_020937.4) | c.5791C>T, p.Arg1931Ter | Het(113/220) | 0.51 | P | P (PVS1, PP3, PP5) | P (PVS1 VS, PM2 M, PP3 Su) | 0.00448 | 0.00101 |
| P055 | AML | 4 | MFD | MSH6 (NM_000179.3) | c.3261del, p.Phe1088SerfsTer2 | Het(109/233) | 0.47 | P | P (PVS1, PM2, PP5) | P (PVS1 VS, PP5- VS, PS3 St, PP3 Su) | NA | 0.00001 |
| P058 | AML | 4 | MFD | MITF (NM_198159.3) | c.1255G>A, p.Glu419Lys | Het(128/263) | 0.49 | P/LP | LP (PM1, PM2, PP3, PP5, BS2) | P (PP5 VS, PS3 St, PM1 M, PP2 Su, PP3 Su) | 0.00111 | 0.00128 |
| P117 | ALL | 10 | MUD | MUTYH (NM_001128425.2) | c.1187G>A, p..Gly396Asp + c.536A>G, p.Tyr179Cys | Het(164/353) Het(118/250) | 0.46 0.47 | P/LP P/LP | LP(PM1, PM2, PP3, PP5, BP1) LP(PM1, PM2, PP3, PP5, BP1) | P (PP V5, PS3 St, PM1 M, PM5 M, PP2 Su) P (PP5 VS, PM1 M, PM2 M, PP2 Su, PP3 Su) | 0.00220 0.00153 | 0.00295 0.00154 |
| P121 | ALL | 11 | MUD | FANCM (NM_020937.4) | c.5101C>T, p.Gln1701Ter | Het(48/111) | 0.43 | P/LP | VUS (PVS1, PP5) | P (PVS1 VS, PP5 VS, PM2 M, BP4 Su) | 0.00823 | 0.00129 |
| P130 | ALL | 16 | MUD | FANCM (NM_020937.4) | c.5101C>T, p.Gln1701Ter | Het(99/205) | 0.48 | P/LP | VUS (PVS1, PP5) | P (PVS1 VS, PP5 VS, PM2 M, BP4 Su) | 0.00823 | 0.00129 |
| P183 | ALL | 1 | MFD | PMS2 (NM_000535.7) | c.325dup, p.Glu109GlyfsTer30 | Het(45/81) | 0.56 | P | P (PVS1, PM2, PP5) | P (PVS1 VS, PM2 M, PP3 Su) | NA | 0.00002 |
| P202 | ALL | 0.9 | MFD | SDHC (NM_003001.5) | c.380A>G, p.His127Arg | Het(93/196) | 0.47 | P/LP | LP (PM1, PM2, PP3, PP5) | P (PP5 St, PM1 M, PM2 M, PM5 M, PP3 Su, BP1 Su) | NA | NA |
| P218 | ALL | 16 | MUD | AR (NM_000044.6) | c.2180G>T, p.Arg727Leu | Hom(163/163) | 1.00 | P | VUS (PM1, PP3, PP5) | LP (PM1 M, PP2 Su, PP3 Su, PP5 Su, BS2 St) | 0.008350 | 0.000950 |

AF: allele frequency; ALL: acute lymphoblastic leukemia; AML: acute myeloid leukemia; CIP: conflicting interpretation of pathogenicity; CLL: chronic lymphocytic leukemia; CML: chronic myeloid leukemia; CMML: chronic myelomonocytic leukemia; Het: Heterozygous; Hom: Homozygous; MDS: myelodysplastic syndrome; MF: myelofibrosis; MM: multiple myeloma; MMFD: mismatched family donor; MMUD: mismatched unrelated donor; MPAL: mixed phenotype acute leukemia; MFD: matched family donor; MUD: matched unrelated donor; NA: not available; P: pathogenic; PCL: plasma cell leukemia; PSV: pathogenic sequence variant; T-ALL: T-cell acute lymphoblastic leukemia; sAML: secondary acute myeloid leukemia; tAML: therapy-related acute myeloid leukemia; UNK: unknown; VAF: variant allele fraction; VUS: variant of unknown significance

^†^Variant known before HSCT

^‡^Criterion level of strength indicated in parentheses: VS: very strong; M: moderate; St: strong; Su; supporting

**Supplementary Table 5.** Demographic data and transplant-related characteristics by germline variant status based on WES data in 386 recipients in Adult cohort 1, Adult cohort 2 and Pediatric cohort who underwent allogeneic hematopoietic stem cell transplantation.

| **Patient characteristics** | ***CHEK2* variant** | **Hematology gene variant** | **Oncology gene variant** | **No variant** |
| --- | --- | --- | --- | --- |
| Number of patients (% of whole cohort) | 44 (11.4) | 14 (3.6) | 24 (6.2) | 304 (78.8) |
| **Gender, N (%)** | | | | |
| Female | 22 (50.0) | 6 (42.9) | 12 (50.0) | 133 (43.8) |
| Male | 22 (50.0) | 8 (57.1) | 12 (50.0) | 169 (55.6) |
| NA | 0 (0) | 0 (0) | 0 (0) | 2 (0.7) |
| **Cohort, N (%)** | | | | |
| Pediatric cohort | 10 (22.7) | 3 (21.4) | 10 (41.7) | 99 (32.6) |
| Adult cohort 1 | 16 (36.4) | 7 (50.0) | 8 (33.3) | 104 (34.2) |
| Adult cohort 2 | 18 (40.9) | 4 (28.6) | 6 (25.0) | 101 (33.2) |
| **Age at HSCT, N (%)** | | | | |
| Age (years, mean ± SD) | 38.3 ± 19.2 | 41.0 ± 18.5 | 32.9 ± 21.8 | 35.1 ± 21.7 |
| ≤ 10 | 6 (13.6) | 1 (7.1) | 6 (25.0) | 68 (22.4) |
| 11 - 20 | 5 (11.4) | 2 (14.3) | 4 (16.7) | 40 (13.2) |
| 21 - 40 | 12 (27.3) | 3 (21.4) | 2 (8.3) | 43 (14.1) |
| > 40 | 21 (47.) | 8 (57.1) | 12 (50.0) | 153 (50.3) |
| **Era of transplantation, N (%)** | | | | |
| 1999-2009 | 17 (38.6) | 6 (42.9) | 8 (33.3) | 132 (43.4) |
| 2010-2020 | 27 (61.4) | 8 (57.1) | 16 (66.7) | 172 (56.6) |
| **Diagnosis group**^†^**, N (%)** | | | | |
| Acute leukemia | 36 (81.8) | 10 (71.4) | 19 (79.2) | 282 (71.4) |
| Lymphoproliferative disease | 4 (9.1) | 1 (7.1) | 2 (8.3) | 42 (11.5) |
| Myeloproliferative disease | 3 (6.8) | 1 (7.1) | 2 (8.3) | 33 (8.9) |
| MDS/JMML | 1 (2.3) | 2 (14.3) | 1 (4.2) | 29 (8.2) |
| **Karnofsky score, N (%)** | | | | |
| 90-100 | 25 (56.8) | 8 (57.1) | 14 (58.3) | 181 (59.5) |
| 0-80 | 1 (2.3) | 2 (14.3) | 1 (4.2) | 6 (2.0) |
| NA | 18 (40.9) | 4 (28.6) | 9 (37.5) | 117 (38.5) |
| **Sorror score, N (%)** | | | | |
| ≥ 3 | 4 (9.1) | 2 (14.3) | 4 (16.7) | 27 (8.9) |
| 0 - 2 | 22 (50.0) | 8 (57.1) | 14 (58.3) | 176 (57.9) |
| NA | 18 (40.9) | 4 (28.6) | 6 (25.0) | 101 (33.2) |
| **Conditioning intensity, N (%)** | | | | |
| MAC | 38 (86.4) | 12 (85.7) | 20 (83.3) | 256 (84.2) |
| RIC | 6 (13.6) | 2 (14.3) | 4 (16.7) | 46 (15.1) |
| NA | 0 (0) | 0 (0) | 0 (0) | 2 (0.7) |
| **Source of transplant, N (%)** | | | | |
| MUD | 18 (40.9) | 9 (64.3) | 10 (41.7) | 139 (45.7) |
| MFD | 25 (56.8) | 5 (35.7)^*^ | 14 (58.3) | 159 (52.3) |
| Haploidentical | 1 (2.3) | 0 (0) | 0 (0) | 6 (2.0) |
| **GVHD prophylaxis, N (%)** | | | | |
| Cyclosporine ± MTX ± Pred ± ATG | 35 (79.5) | 12 (85.7) | 20 (83.3) | 232 (76.3) |
| Cyclosporine + MMF | 8 (18.2) | 2 (14.3) | 4 (16.7) | 63 (20.7) |
| Post-transplantation cyclophosphamide | 1 (2.3) | 0 (0) | 0 (0) | 4 (1.3) |
| No prophylaxis or NA | 0 (0) | 0 (0) | 0 (0) | 5 (1.6) |
| **Viremia/bacteremia, N (%)** | | | | |
| Yes | 12 (27.3) | 6 (42.9) | 6 (25.0) | 75 (24.7) |
| No | 14 (31.8) | 4 (28.6) | 12 (50.0) | 128 (42.1) |
| NA | 18 (40.9) | 4 (28.6) | 6 (25.0) | 101 (33.2) |

ATG: anti-thymocyte globulin; GVHD: graft-versus-host disease; HSCT: hematopoietic stem cell transplantation; JMML: juvenile myelomonocytic leukemia; MAC: myeloablative conditioning; MDS: myelodysplastic syndrome; MMF: mycophenolate mofetil; MFD: matched family donor; MTX: methotrexate; MUD: matched unrelated donor; NA: not available; Pred: prednisolone; RIC: reduced intensity conditioning; WES: whole exome sequencing.

^†^The diagnoses categorized as acute leukemia include acute myeloid leukemia, secondary acute myeloid leukemia, therapy-related acute myeloid leukemia, acute lymphoblastic leukemia, mixed phenotype acute leukemia. The diagnoses in the lymphoproliferative disease group include malignant myeloma, plasma cell leukemia, non-Hodgkin’s lymphoma, T-cell prolymphocytic leukemia and chronic lymphocytic leukemia. The diagnoses in the myeloproliferative disease group include chronic myelomonocytic leukemia, chronic myeloid leukemia, myeloproliferative neoplasm, myelofibrosis, mastocytosis.

^*^ P< 0.05 when compared to the *CHEK2*, Oncology Panel, and no-variant groups by Fisher's exact tests.

**Supplementary Table 6.** Demographic data, transplant-related characteristics and outcome of 14 patients with a germline P/LP variant in the Hematology panel in the WES data set who underwent allogeneic hematopoietic stem cell transplantation.

| **Patient** | **Gender** | **Age at HSCT (years)** | **Diagnosis** | **Donor type** | **Gene** | **Conditioning (RIC/MAC)** | **Acute GVHD** | **Chronic GVHD** | **Alive at day 100 post-HSCT** | **Alive at end of follow-up** | **Cause of death in deceased patients** |
| --- | --- | --- | --- | --- | --- | --- | --- | --- | --- | --- | --- |
| A038 | Male | 64 | AML | MMUD | DDX41 | MAC | No | Yes | Yes | No | Relapse |
| A039 | Male | 55 | ALL | MFD | RUNX1 | MAC | Yes | No | Yes | No | Relapse |
| A041 | Female | 59 | MDS/MPN | MUD | TERT | MAC | Yes | Not applicable | No | No | Treatment-related toxicity |
| A042 | Female | 56 | MM | MUD | POT1 | RIC | Yes | Yes | Yes | Yes | - |
| A063 | Male | 38 | AML | MUD | ERCC6L2 | MAC | No | No | Yes | No | Relapse |
| A095^†^ | Female | 41 | MDS | MUD | GATA2 | MAC | Yes | No | Yes | Yes | - |
| A102^†^ | Female | 32 | MDS | MMUD | GATA2 | MAC | No | Yes | Yes | No | Treatment-related toxicity |
| V009 | Female | 44 | AML | Not available | GATA2 | MAC | No | No | Yes | Yes | - |
| V092 | Male | 63 | AML | Not available | SAMD9 | RIC | No | Yes | Yes | Yes | - |
| V102 | Male | 36 | AML | Not available | ANKRD26 | MAC | No | Yes | Yes | Yes | - |
| V117 | Female | 48 | ALL | Not available | ANKRD26 | MAC | Yes | No | Yes | Yes | - |
| P169 | Male | 5 | ALL | MUD | TP53 | MAC | Yes | Not applicable | No | No | Treatment-related toxicity |
| P151 | Male | 14 | ALL | MUD | TP53 | MAC | No | No | Yes | No | Relapse |
| P184^†^ | Male | 12 | AML | MUD | GATA2 | MAC | Yes | No | Yes | No | Relapse |

ALL: acute lymphoblastic leukemia; AML: acute myeloid leukemia; GVHD: graft-versus-host disease; HSCT: hematopoietic stem cell transplantation; MAC: myeloablative conditioning; MDS: myelodysplastic syndrome; MFD: matched family donor; MM: multiple myeloma; MMUD: mismatched unrelated donor; MPN: myeloproliferative neoplasm; MUD: matched unrelated donor; RIC: reduced intensity conditioning.

^†^Variant known before HSCT

**Supplementary Table 7**. Pathogenic or likely pathogenic gene variants by WES data found in donors in Adult cohort 2 and Pediatric cohort grouped according to the American College of Medical Genetics and Genomics (ACMG) classification. The donors analyzed were matched family donors.

| **Donor** | **Gene  (Transcript)** | **Panel** | **Base change,  Amino acid change** | **Genotype (calls/coverage)** | **VAF** | **Clinvar** | **Intervar** | **AF (all)** | **AF (Fin)** |
| --- | --- | --- | --- | --- | --- | --- | --- | --- | --- |
| D1 | SAMD9 (NM_001193307.1) | Hematology | c.1030C>T, p.Arg344Ter | Het(11/28) | 0.39 | P | VUS | 0.00004 | NA |
| D2 | DDX41  (NM_016222.4) | Hematology | c.3G>A, p.Met1Ile | Het(9/12) | 0.75 | P/LP | VUS | 0.00007 | 0.00005 |
| D3 | ANKRD26 (NM_014915.3) | Hematology | c.4633G>T, p.Glu1545Ter | Het(10/33) | 0.3 | UNK | LP | 0.0000041 | NA |
| D4 | AR  (NM_000044.6) | Oncology | c.2180G>T, p.Arg727Leu | Hom(12/12) | 1 | P | VUS | 0.00093 | 0.00837 |
| D5 | AR  (NM_000044.6) | Oncology | c.2180G>T, p.Arg727Leu | Hom(10/10) | 1 | P | VUS | 0.00093 | 0.00837 |
| D6 | FANCM (NM_020937.4) | Oncology | c.5101C>T, p.Gln1701Ter | Het(22/38) | 0.58 | P/LP | VUS | 0.00129 | 0.00821 |
| D7 | FANCM (NM_020937.4) | Oncology | c.5101C>T, p.Gln1701Ter | Het(14/34) | 0.41 | P/P | VUS | 0.00129 | 0.00821 |
| D8 | FANCM (NM_020937.4) | Oncology | c.5101C>T, p.Gln1701Ter | Het(21/42) | 0.5 | P/LP | VUS | 0.00129 | 0.00821 |
| D9 | FANCM (NM_020937.4) | Oncology | c.5101C>T, p.Gln1701Ter | Het(23/52) | 0.44 | P/LP | VUS | 0.00129 | 0.00821 |

AF: allele frequency; Het: heterozygous; Hom: homozygous; LP: likely pathogenic; NA: not available; P: pathogenic; UNK: unknown; VAF: variant allele fraction; VUS: variant of unknown significance; WES: whole exome sequencing.

**Supplementary Table 8.** Multivariable models of transplant outcomes of 877 recipients who underwent allogeneic hematopoietic stem cell transplantation. Cox regression analysis for (A) risk of death, (B) risk of relapse and (C) risk of non-relapse mortality based on HSCT outcome risk factors and *CHEK2* variant status by WES and SNP data. *P*-values below 0.05 are considered statistically significant and are highlighted in bold.

| **A) Risk of death** | **Hazard ratio** | **95 % CI** | **p-value** |
| --- | --- | --- | --- |
| Patient's age (10 years) | 1.21 | 1.10 - 1.33 | **<.001** |
| Patient's gender (female vs. male) | 0.87 | 0.68 - 1.11 | 0.265 |
| Donor's gender (female vs. male) | 0.98 | 0.76 - 1.26 | 0.887 |
| Transplantation era | 1.04 | 0.84 - 1.27 | 0.736 |
| Diagnosis (acute leukemia vs. other) | 1.40 | 1.06 - 1.86 | **0.017** |
| Conditioning (RIC vs. MAC) | 1.25 | 0.92 - 1.70 | 0.155 |
| Source of Transplant (MFD vs. MUD) | 0.61 | 0.47 - 0.78 | **<.001** |
| *CHEK2* variant (patient) | 1.01 | 0.69 - 1.46 | 0.973 |
| *CHEK2* variant (donor) | 0.80 | 0.50 - 1.28 | 0.357 |

| **B) Risk of relapse** | **Hazard ratio** | **95 % CI** | **p-value** |
| --- | --- | --- | --- |
| Patient's age (10 years) | 1.07 | 0.97 - 1.19 | 0.192 |
| Patient's gender (female vs. male) | 1.20 | 0.91 - 1.57 | 0.194 |
| Donor's gender (female vs. male) | 1.04 | 0.79 - 1.37 | 0.805 |
| Transplantation era | 0.83 | 0.65 - 1.04 | 0.104 |
| Diagnosis (acute leukemia vs. other) | 0.77 | 0.56 - 1.06 | 0.114 |
| Conditioning (RIC vs. MAC) | 1.30 | 0.93 - 1.82 | 0.125 |
| Source of Transplant (MFD vs. MUD) | 0.67 | 0.50 - 0.89 | **0.006** |
| *CHEK2* variant (patient) | 1.19 | 0.80 - 1.78 | 0.399 |
| *CHEK2* variant (donor) | 0.77 | 0.45 - 1.31 | 0.332 |

| **C) Risk of non-relapse mortality** | **Hazard ratio** | **95 % CI** | **p-value** |
| --- | --- | --- | --- |
| Patient's age (10 years) | 1.30 | 1.11 - 1.51 | **<.001** |
| Patient's gender (female vs. male) | 0.77 | 0.53 - 1.14 | 0.193 |
| Donor's gender (female vs. male) | 0.87 | 0.58 - 1.30 | 0.496 |
| Transplantation era | 1.28 | 0.92 - 1.78 | 0.147 |
| Diagnosis (acute leukemia vs. other) | 1.37 | 0.90 - 2.10 | 0.144 |
| Conditioning (RIC vs. MAC) | 1.32 | 0.83 - 2.10 | 0.246 |
| Source of Transplant (MFD vs. MUD) | 0.63 | 0.42 - 0.93 | **0.021** |
| *CHEK2* variant (patient) | 0.95 | 0.52 - 1.74 | 0.876 |
| *CHEK2* variant (donor) | 0.79 | 0.37 - 1.72 | 0.558 |

MAC: myeloablative conditioning; MFD: matched family donor; MUD: matched unrelated donor; RIC: reduced intensity conditioning; SNP: single nucleotide polymorphism; WES: whole exome sequencing.


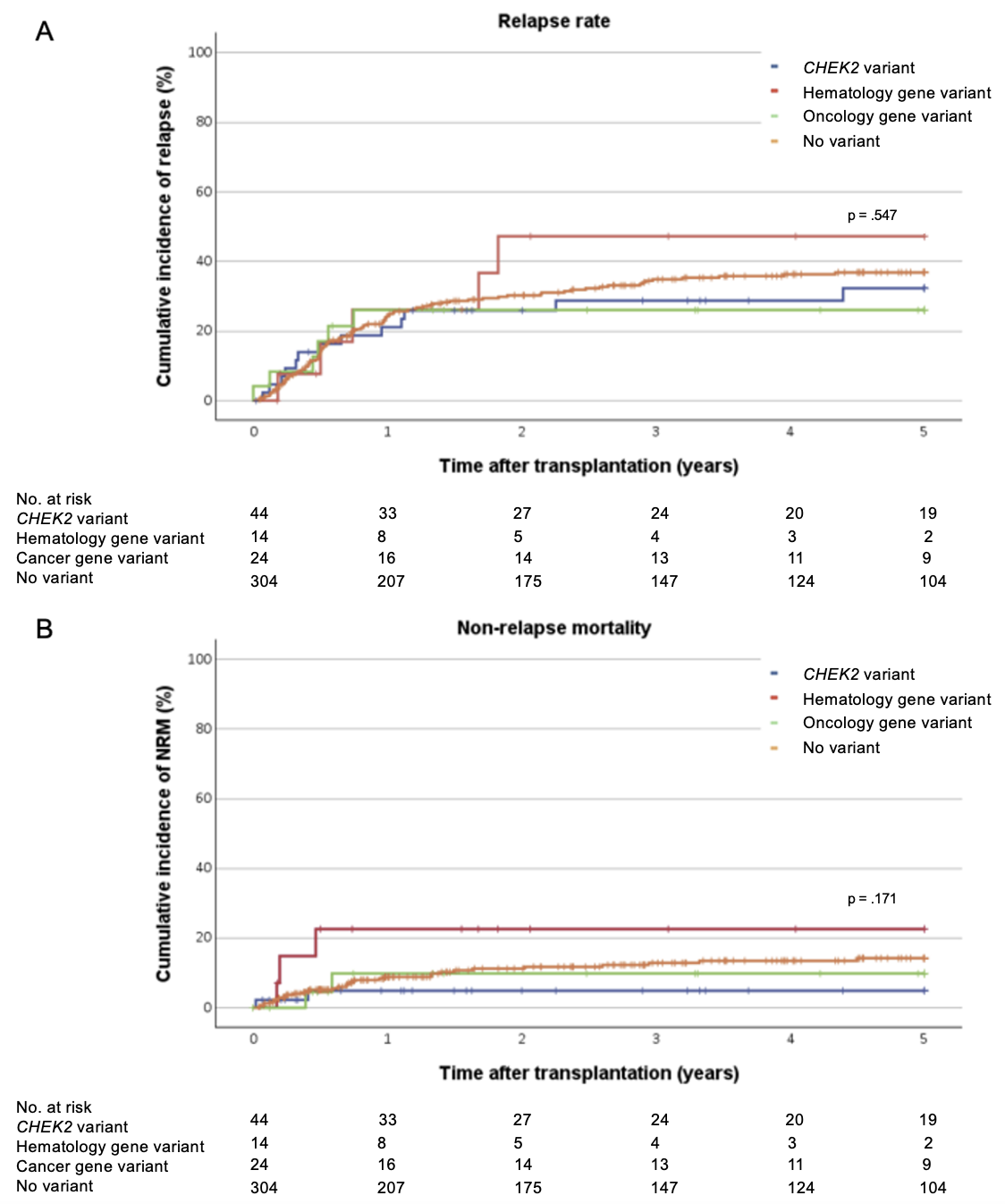


**Supplementary Figure 1.** Transplant outcomes by germline gene variant status by whole exome sequencing data in Adult cohort 1, Adult cohort 2 and Pediatric cohort. (A) Cumulative incidence curves for relapse rate and (B) cumulative incidence curves for non-relapse mortality. Recipients with a variant in a Hematology panel gene demonstrated a tendency toward increased relapse rates and non-relapse mortality compared to those without any variant. *P*-values provided using the log-rank test did not reach statistical significance. Tick marks indicate censored data.


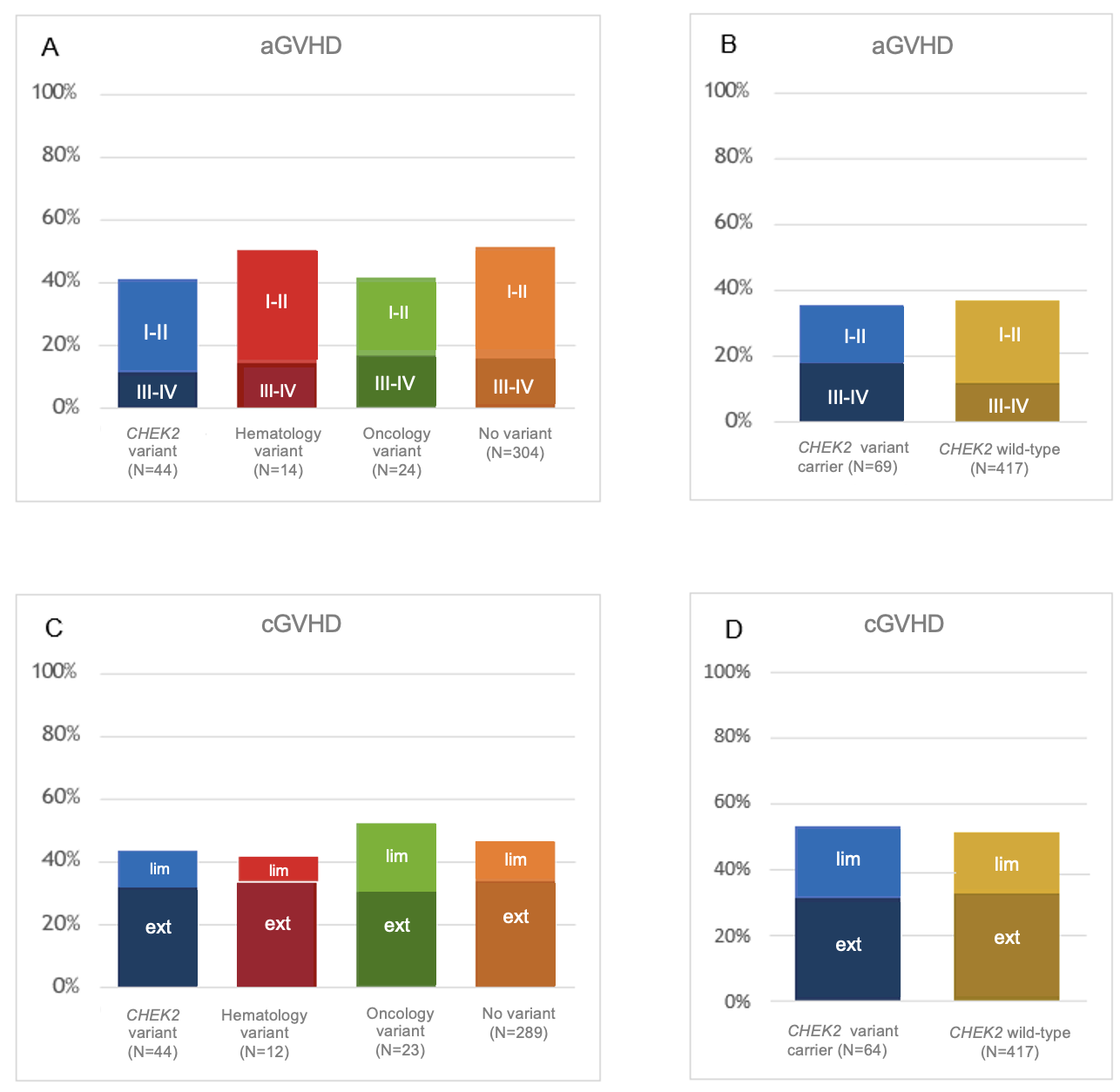


**Supplementary Figure 2.** Rates of acute graft-versus-host disease (aGVHD) and chronic graft-versus-host disease (cGVHD) by germline variant status in recipients and donors. The rates of aGVHD divided for stages I-II and III-IV^2^ are given (A) by *CHEK2* variant, Hematology gene variant, Oncology gene variant or no variant status of recipients based on WES data; (B) by *CHEK2* variant status of donors based on WES and SNP data. The rates of cGVHD, classified as extensive (ext) and limited (lim) according to the Seattle classification^3^, are given (C) by *CHEK2* variant, Hematology gene variant, Oncology gene variant or no variant status of recipients based on WES data, (D) by *CHEK2* variant status of donors based on WES and SNP data. WES: whole exome sequencing; SNP: single nucleotide polymorphism.


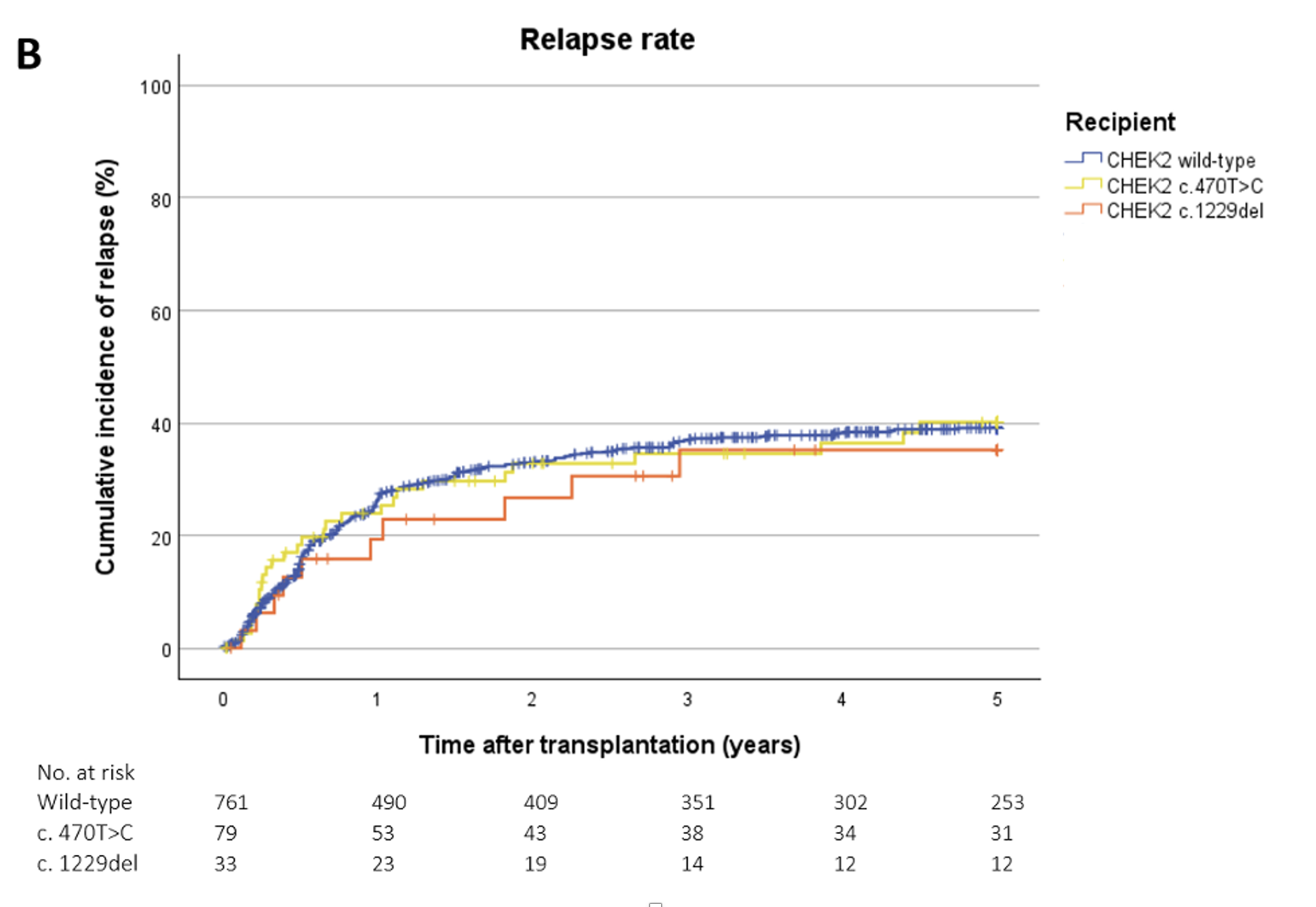


A

p = .907

p = .604

B


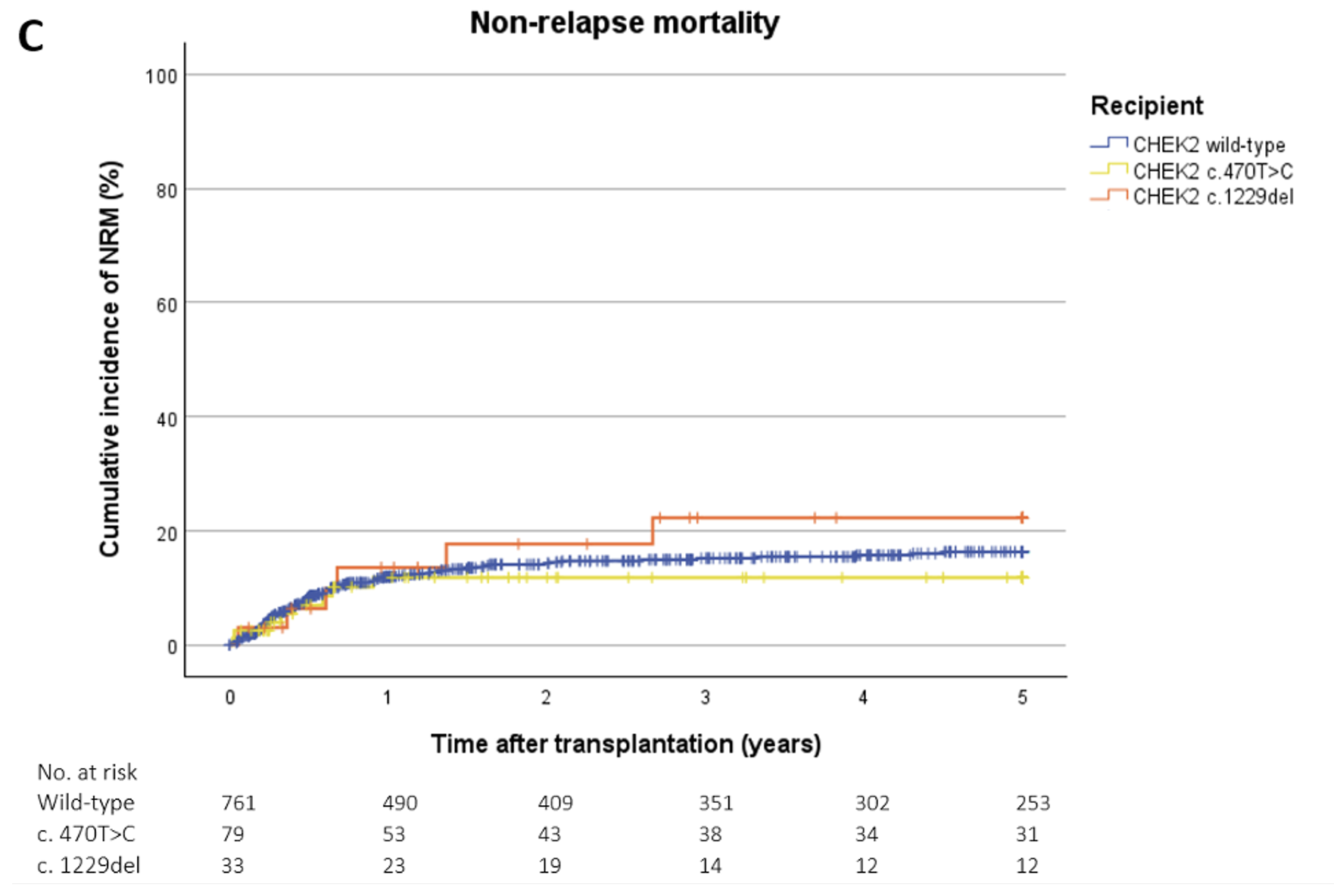


p = .498

p = .469

**Supplementary Figure 3.** Transplant outcomes by recipient *CHEK2* variant status in Adult cohort 1, Adult cohort 2, Adult cohort 3 and Pediatric cohort based on whole exome sequencing and single nucleotide polymorphism data. Kaplan-Meier curve for (A) cumulative incidence curves for relapse rate and (B) non-relapse mortality, comparing patients with and without the *CHEK2* variants c.1229del, p. Cys410SerfsTer4 and c.470T>C, p.Ile157Thr. *P*-values provided using the log-rank test did not reach statistical significance. Tick marks indicate censored data.

A


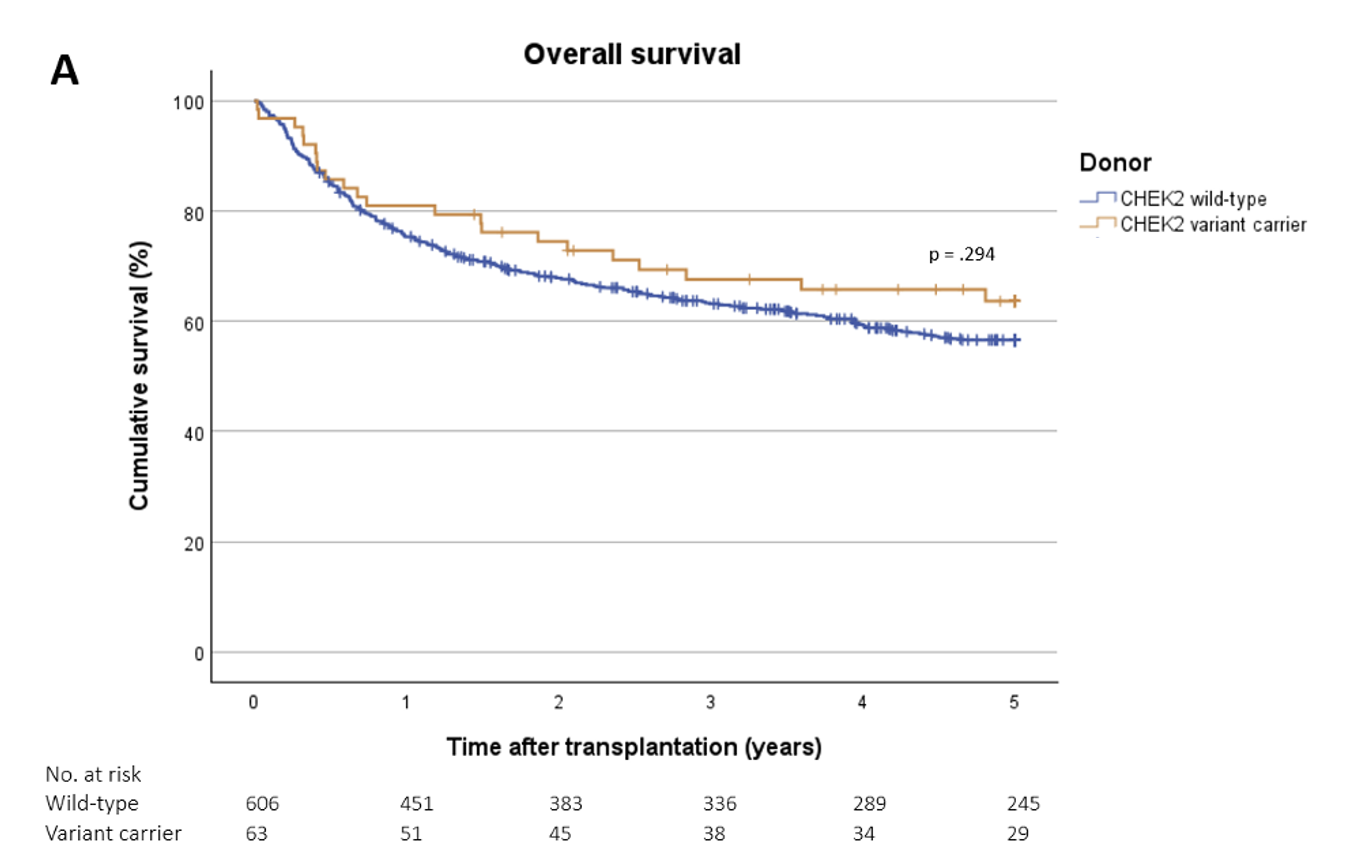


B


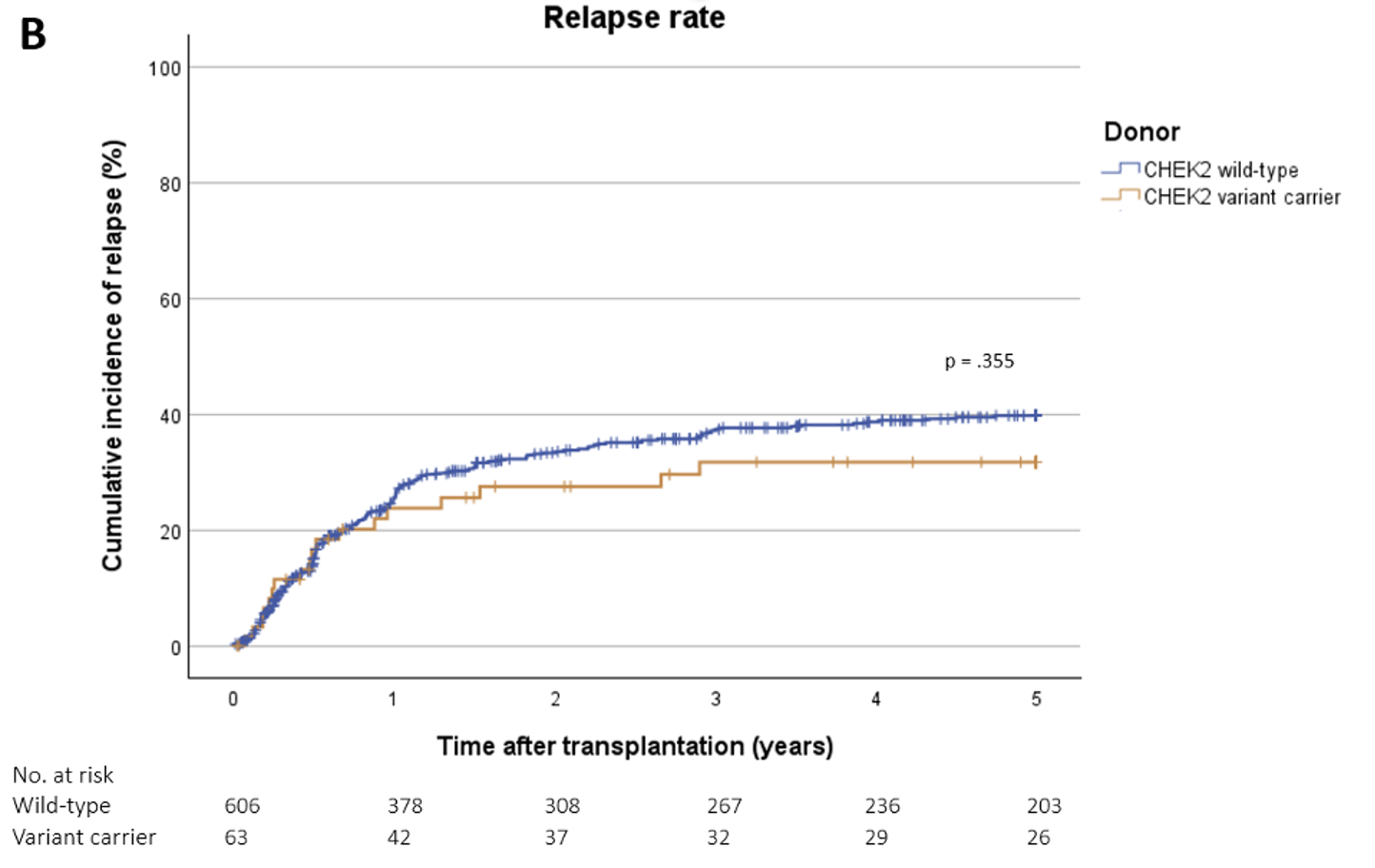


C


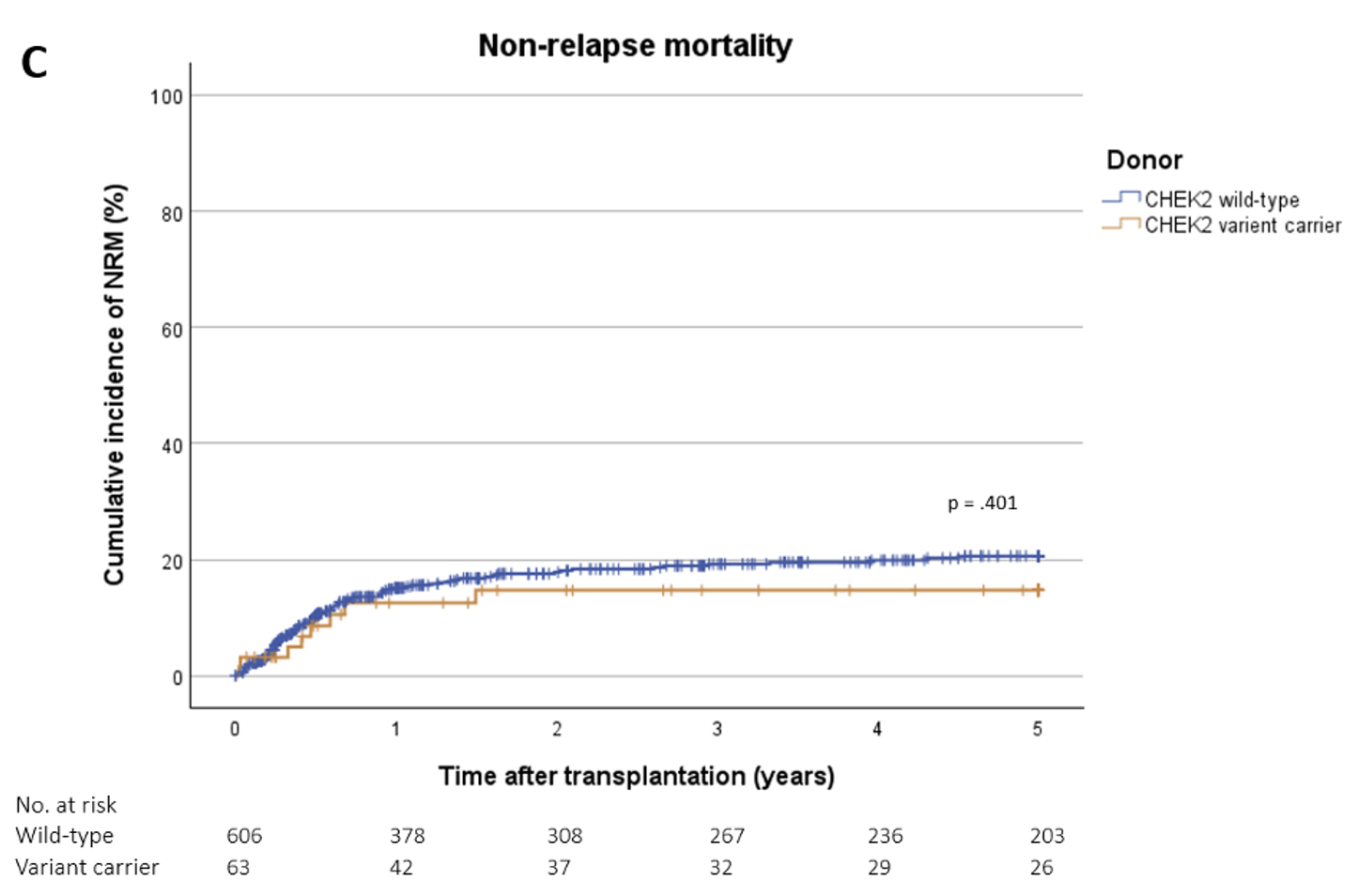


**Supplementary Figure 4.** Transplant outcomes by donor *CHEK2* variant status in Adult cohort 1, Adult cohort 2, Adult cohort 3 and Pediatric cohort based on whole exome sequencing and single nucleotide polymorphism data. Kaplan-Meier curve for (A) overall survival, (B) cumulative incidence curves for relapse rate and (C) non-relapse mortality outcomes, by donor variant status with and without *CHEK2* variant c.1229del, p. Cys410SerfsTer4 or c.470T>C, p.Ile157Thr. *P*-values provided using the log-rank test did not reach statistical significance. Tick marks indicate censored data.

**REFERENCES**

1. Nihtilä J, Salmenniemi U, Itälä-Remes M, Crossland RE, Gallardo D, Bogunia-Kubik K, et al. Donor genetic determinant of thymopoiesis, rs2204985, and stem cell transplantation outcome in a multipopulation cohort. Human Immunology. 2024 May;85(3):110791.
2. Przepiorka D, Weisdorf D, Martin P, Klingemann HG, Beatty P, Hows J, et al. 1994 Consensus Conference on Acute GVHD Grading. Bone Marrow Transplant. 1995 Jun;15(6):825–8.
3. Lee SJ, Vogelsang G, Flowers MED. Chronic graft-versus-host disease. Biology of Blood and Marrow Transplantation. 2003 Apr 1;9(4):215–33.
